# A Phase 1b Randomized, Controlled, Double-Blinded Dosage-Escalation Trial to Evaluate the Safety, Reactogenicity and Immunogenicity of an Adenovirus Type 35 Based Circumsporozoite Malaria Vaccine in Burkinabe Healthy Adults 18 to 45 Years of Age

**DOI:** 10.1371/journal.pone.0078679

**Published:** 2013-11-11

**Authors:** Alphonse Ouédraogo, Alfred B. Tiono, Désiré Kargougou, Jean Baptiste Yaro, Esperance Ouédraogo, Youssouf Kaboré, David Kangoye, Edith C. Bougouma, Adama Gansane, Noelie Henri, Amidou Diarra, Souleymane Sanon, Issiaka Soulama, Amadou T. Konate, Nora L. Watson, Valerie Brown, Jenny Hendriks, Maria Grazia Pau, Isabella Versteege, Edison Wiesken, Jerald Sadoff, Issa Nebie, Sodiomon B. Sirima

**Affiliations:** 1 Centre National de Recherche et de Formation sur le Paludisme, Ouagadougou, Burkina Faso; 2 The EMMES Corporation, Rockville, Maryland, United States of America; 3 Crucell Holland BV, Leiden, The Netherlands; 4 Groupe d’action et de Recherche en Santé, Ouagadougou, Burkina Faso; Kenya Medical Research Institute (KEMRI), Kenya

## Abstract

**Background:**

Ad35.CS.01 is a pre-erythrocytic malaria candidate vaccine. It is a codon optimized nucleotide sequence representing the *P. falciparum* circumsporozoite (CS) surface antigen inserted in a replication deficient Adenovirus 35 backbone. A Phase 1a trial has been conducted in the USA in naïve adults and showed that the vaccine was safe. The aim of this study is to assess the safety and immunogenicity of ascending dosages in sub Saharan Africa.

**Methods:**

A double blind, randomized, controlled, dose escalation, phase Ib trial was conducted in a rural area of Balonghin, the Saponé health district (Burkina Faso). Forty-eight healthy adults aged 18-45 years were randomized into 4 cohorts of 12 to receive three vaccine doses (day 0, 28 and 84) of 10^9^, 10^10^, 5X10^10^, 10^11^ vp of Ad35.CS.01 or normal saline by intra muscular injection. Subjects were monitored carefully during the 14 days following each vaccination for non serious adverse events. Severe and serious adverse events were collected throughout the participant study duration (12 months from the first vaccination). Humoral and cellular immune responses were measured on study days 0, 28, 56, 84, 112 and 140.

**Results:**

Of the forty-eight subjects enrolled, forty-four (91.7%) received all three scheduled vaccine doses. Local reactions, all of mild severity, occurred in thirteen (27.1%) subjects. Severe (grade 3) laboratory abnormalities occurred in five (10.4%) subjects. One serious adverse event was reported and attributed to infection judged unrelated to vaccine. The vaccine induced both antibody titers and CD8 T cells producing IFNγ and TNFα with specificity to CS while eliciting modest neutralizing antibody responses against Ad35.

**Conclusion:**

Study vaccine Ad35.CS.01 at four different dose levels was well-tolerated and modestly immunogenic in this population. These results suggest that Ad35.CS.01 should be further investigated for preliminary efficacy in human challenge models and as part of heterologous prime-boost vaccination strategies.

**Trial Registration:**

ClinicalTrials.gov NCT01018459 http://clinicaltrials.gov/ct2/show/NCT01018459

## Introduction

Malaria is one of the most prevalent infections in tropical and subtropical areas throughout the world. An estimated 3.3 billion people were at risk of malaria in 2010 and regions of highest risk concentrated in sub-Saharan Africa[1]. Eighty-one percent of cases and 91% of deaths were estimated to have occurred in the World Health Organization African region, predominantly among children under five years of age and pregnant women[1]. Drug-resistant parasites and insecticide-resistant vectors increasingly pose challenges for malaria control programs. Failure of current control measures calls for an accelerated development of malaria vaccine as a potentially effective additional tool in the arsenal against malaria. No vaccine has been licensed to protect against malaria. In recent years, the effort to develop an effective malaria vaccine has resulted in a number of malaria vaccine candidates reaching the stage of testing in malaria-exposed populations. 

Malaria parasites have a life cycle consisting of several developmental stages. Each of these stages is able to induce specific immune responses against the corresponding stage-specific antigens. A number of vaccines have been tested that target molecules expressed at different stages of the parasite’s life cycle. In this clinical trial, a pre-erythrocyte malaria vaccine that expresses the CS protein using a replication deficient Ad35 as a viral vector was studied. Ad35.CS.01 is a malaria vaccine for which a codon optimized nucleotide sequence representing the *P. falciparum* circumsporozoite (CS) surface antigen is inserted in a replication deficient Adenovirus 35 backbone. 

The CS protein is the major surface protein of *P. falciparum* sporozoite. CS is a 350 residue protein and is thought to have multiple important functions. One function of the CS protein is to mediate sporozoite attachment to heparan sulfate proteoglycans in the liver of the mammalian host[2-7]. Other proposed functions involve sporozoite binding to mosquito salivary gland [8,9], sporozoite gliding motility [10], a form of substrate dependant cell locomotion characteristic of *Apicomplexa*, as well as inhibition of host intracellular protein synthesis by binding to ribosomes in hepatocytes[11]. 

Given the important function mediated by CS protein during the malaria parasite cycle, the CS protein has become one of the ideal targets for vaccine design. CS-based vaccines have been shown to induce antibody and T cell responses that both block and neutralize sporozoites from invading liver cells and/or destroy sporozoite-infected liver cells[12,13]. Hence, large-scale deployment of an effective vaccine against the pre-erythrocytic stages of *P. falciparum* could potentially prevent millions of cases of malaria disease. Clinical trials of various CS-based vaccines have been conducted in human volunteers and have been shown to elicit antigen- specific immune responses [14-16]. The positive results obtained in clinical trials with CS-based vaccines have encouraged efforts to enhance potential efficacy through a variety of methods to improve vaccine delivery and immunogenicity of these vaccines. 

Recombinant adenovirus vectors have been widely evaluated as vectors for therapeutic gene transfer, oncolytic viral vectors and vaccine vectors. Because of the extensive knowledge of its genome, interactions, and safety profile, a replication-deficient adenovirus was selected as the vector system to deliver the above mentioned codon optimized CS- protein. Ad35 was selected in preference to Ad5 for the CS-based vaccine construction because of its low seroprevalence worldwide. In a sero-epidemiologic study, neutralizing antibody titers to Ad35 were much lower than Ad5 titers, most notably in sub-Saharan Africa, suggesting that a vaccine based on Ad35 vector might not be subject to the problem of limited effectiveness of Ad5- vectored vaccines due to anti-vector neutralizing pre-existing antibody activity[17]. 

In the genome structure of the attenuated replication deficient Ad35.CS.01, the E1 region is replaced with the CS gene. Due to the deletion of the E1 gene, which codes for a protein required for trans- activation of viral gene, the Ad35 vaccine vector is unable to complete viral gene transcription process after infecting host cells; thus, no viral proteins are synthesized, no viral particles are produced and therefore, Ad35.CS.01 is replication deficient. The inserted CS gene is driven by a CMV promoter and is expressed constitutively. 

It is believed that Ad35.CS.01 will stimulate the immune system to induce CS-specific humoral or cell mediated immunity by infecting host cells, including professional antigen presenting cells (APCs), such as dendritic cells[18], macrophages or B cells. The encoded CS protein of interest is then synthesized by the host cell’s protein expression machinery. The CS protein may potentially be processed further via MHC class I antigen processing and presentation pathway and presented in the context of MHC class I molecules. By recognizing cell surface MHC class I/peptide complex, CD8 T cells are activated, proliferate and further mature into effectors that exert either cytolytic function or cytokine secretion. Vaccines based on these non-replicating adeno-vectors are capable of inducing antigen specific CD4 and CD8 positive T cells in humans [19,20] . Vaccines inducing CS antigen specific CD4 and CD8 T cells were shown to be protective in mouse malaria models in the late 1980s [21,22], and recently CD8 T cells were implicated in destroying parasite infected liver cells and thereby preventing disease after infection [23]. In addition, these adenovectored vaccines are capable of inducing antibody responses in humans against their inserted transgenes[19,20] . It is anticipated that the antibody response will be primarily responsible for binding to the CS protein on the surface of sporozoites that enter the host’s liver cells. The combined action of both antibody and T cell responses is expected to ultimately prevent the parasite from producing clinical disease. 

The Ad35.CS.01 Phase 1a study in the U.S. found the vaccine safe in malaria naïve adults. This clinical trial evaluated the safety, reactogenicity and immunogenicity of different doses of Ad35.CS.01 in healthy semi immune adults.

## Materials and Methods

The protocol for this trial and supporting CONSORT checklist are available as supporting information; see Checklist S1 and Protocol S1. 

### Trial site and population

The study was carried out by the Centre National de Recherche et de Formation sur le Paludisme (CNRFP) in the malaria vaccine clinical trials center located in the village of Balonghin, in the Saponé health district in the Bazèga province from April 2010 to December 2011. The clinical trial center is equipped and staffed to undertake clinical trial phases 1b through 2b in compliance with national and international standards and requirements. An ambulance was available in Balonghin 24 h per day throughout the 1 year follow-up period. Sapone district hospital and the National Teaching Hospital in Ouagadougou both serve as referral hospitals for study participants requiring specialized care. 

This rural area is situated 50 km south east of Ouagadougou, the capital city of Burkina Faso in the Sudan-Sahelian eco-climatic zone. In this area, malaria transmission is markedly seasonal and intense during the rainy season from June to October. The entomological inoculation rate is above 40 infective bites/person/night during the intense transmission season and drops to 0 during the low season (dry season). The main malaria vectors are *Anopheles gambiae, An. Arabiensis and An. funestus*. *P falciparum* accounts for about 90% of malaria infections in children below the age of 5 during the high transmission season.

### Study design and objectives

The study was a single-center, double-blinded, dosage-escalation, randomized phase Ib clinical trial designed to assess the safety of four ascending dosages of the Ad35.CS.01 malaria candidate vaccine. (www.clinicaltrials.gov NCT01018459). Trial participants were recruited among healthy male and female adults living in Balonghin and neighbour villages. The primary objective was to assess the safety and reactogenicity of four ascending dosages of the Ad35.CS.01 malaria candidate vaccine among healthy, semi-immune, subjects given in 3 doses at 0, 1 and 3 months by intramuscular injection. Ten subjects received vaccine at each of the following ascending dosage levels: 10^9^ viral particles (vp)/mL, 10^10^ vp/mL, 5 x 10^10^ vp/mL and 10^11^vp/mL. Two subjects in each group received normal saline as placebo control. Dosage escalation preceded only after review of the 14-day safety data of the prior dosage level by the Safety Monitoring Committee (SMC).

### Screening and enrolment

Initially, an educational process was instituted to represent several levels of authority starting from the civil authorities to the local village leadership and down to small groups and ultimately the individual. From the up to date database of the Demographic Surveillance System (DSS) of the population in the study area, a list of all adults 18-45 years old from the village of Balonghin and neighbour villages has been drawn. Subjects were visited at home by field workers who explained the study to them. They were then invited to the vaccinology unit based at Balonghin village to receive more information about the study. Those who provided signed informed consent were screened for study eligibility. Individuals were eligible for inclusion in the trial if they were found to be healthy at a general medical examination, indicated their intention to reside in the village for the duration of the trial (12 months) and gave written informed consent. 

Exclusion criteria included: (i) abnormal screening laboratory values; (ii) positive serology for human immunodeficiency virus (HIV), hepatitis C virus (HCV), or hepatitis B surface antigen (HBsAg); (iii) concurrent participation in other investigational protocols or receipt of an investigational product within the previous 30 days or planned receipt of an investigational product within 28 days following the last immunization dose. A total of 48 adults meeting the inclusion and exclusion criteria were enrolled, assigned a unique identification number and given an identity card.

### Randomization

Clinic staff randomized eligible subjects using the enrollment module of the EMMES Corporation’s AdvantageEDC^SM^ electronic data capture system, which for this study was designed to assign vaccine and placebo in a 5:1 ratio. All study staff were blinded to assignment with the exceptions of the investigational pharmacist and the unblinded vaccinator. 

### Study vaccines and vaccination

Ad35.CS.01 vaccine product was produced in complementing PER.C6^®^ cells by Crucell Holland BV, Leiden under DMID-supported contract N01-AI05421, NIAID, NIH, Bethesda. The *P. falciparum* CS gene insert is a synthetic, mammalian-codon-optimized insert encoding a CS protein as previously described[24]. Each vial of drug product (Ad35.CS.01 vaccine) contains Ad 35.CS.01, Tris, Magnesium Chloride, Sodium Chloride, Sucrose, and PolySorbate-80 (non-animal source). 

The diluent used to dilute the drug product to the intended dosages for the clinical study. The diluent contains Tris, Magnesium Chloride, Sodium Chloride, Sucrose, and PolySorbate-80 (non-animal source). 

Vaccine and diluent were shipped from Crucell. The normal saline for use as placebo was shipped from the DMID Clinical Agent Repository at Fisher BioServices. 

Ad35.CS.01 was stored at ≤ - 65°C. Storage was in a monitored and alarmed freezer in the investigational pharmacy. The diluent was refrigerated at 2 to 8°C. The normal saline, supplied in single dose vials, refrigerated at 2 to 8°C.

### Blinding

After randomization in AdvantageEDC, the pharmacist prepared the injection of vaccine or placebo control according to the treatment code list securely maintained in the pharmacy. No subjects or study personnel were present at the time of preparation. The pharmacist delivered the syringe masked with tape to an unblinded vaccinator who administered the study product. Safety and reactogenicity outcomes were evaluated by study staff members who were blinded to treatment assignment. 

### Assessment of study outcomes: Safety and Reactogenicity

During each visit, a targeted physical exam was performed according to the medical history. Following each vaccination, subjects were observed in the clinic for a minimum of 30 minutes to record any immediate local or systemic adverse reactions. Field workers then visited the subjects at home to collect the 14 days solicited or unsolicited local and systemic reactions. After the 14 days follow-up following the third vaccination, participants were followed up on or around 112, 140, 252 and 365 days after the first vaccine dose was given.

Solicited local adverse events at the injection site included pain, tenderness, erythema, induration and ecchymosis. The severity of pain was assessed based on duration and intensity. Grade 1 pain was defined as mild pain, does not interfere with activity; grade 2 pain was moderate, interferes with activity; grade 3 pain was severe, prevents daily activity.

Other local reactions, erythema, induration and ecchymosis were assessed by their size and the duration. Severity was graded based on the measurement of the greatest surface diameter in cm. Grading was as follows: 1= >0 - 5 cm and does not interfere with activity, 2= 5.1 - 10 cm or interferes with activity, 3= >10 cm or prevents daily activity. 

Solicited systemic reactions included fever, headache, malaise, myalgia, chills, nausea and vomiting. An axillary temperature of 37.5°C and over is considered fever in adults. Axillary temperature was measured using a digital thermometer and fever evaluated according to the grading scale: 1= 37.8 - 38.4°C, 2= 38.5 - 38.9°C and 3= >38.9°C.The severity of systemic reactions was assessed based on duration and intensity. The grade 1 (mild) is when a symptom doesn’t interfere with activity; grade 2 (moderate) when a symptom interferes with activity, grade 3 (severe) prevents daily activity. Any other symptoms not included in the targeted symptoms list above were recorded as non-solicited symptoms by the investigators. 

Solicited and unsolicited systemic reactions/events were monitored at each contact from day 0 to day 112. The occurrence of serious adverse events was monitored throughout the entire study period. A serious adverse event is defined as an adverse event meeting one of the following conditions: death, life threatening, requiring hospitalization, congenital anomaly/birth defect, any other important medical event.

Blood and urine samples were obtained at screening and on days 7, 35 and 91 to monitor biological safety of the vaccine. Biological safety tests included hematology [hemoglobin (Hgb), white blood cell count (WBC) with machine differential, absolute neutrophil count (ANC), and platelet count], blood biochemistry [glucose, electrolytes (sodium, potassium), alanine aminotransferase (ALT), aspartate aminotransferase (AST), and creatinine] and urinalysis testing included a dipstick for blood and proteins. HIV serology was assessed at the screening and the close out visit. 

A thick and thin film was prepared and a rapid diagnosis test for malaria was performed if a trial subject presented with an axillary temperature ≥37.5°C or history of fever within the prior 24 hours. The rapid diagnostic test results were used to guide prompt treatment while awaiting the results of the slide examination. Subjects diagnosed with uncomplicated malaria were treated with 6 equal doses of Coartem® at 0, 8, 24, 36, 48 and 60 hours according to the national guidelines for care in Burkina Faso. All adverse events were followed up to resolution or stabilization.

### Assessment of study outcomes: immunogenicity

To assess both humoral and cell-mediated immune responses to the vaccine, 50 mL of blood was drawn from subjects on the following study days: 0, 28, 56, 84, 112, 140 days. From the 50 mL blood for immunological responses, 10mL was used for serum isolation and subsequent humoral assays, and 40 mL blood was used for cellular assays: 30mls for PBMC isolation and 10 ml for whole blood stimulation followed by cytokine ELISA. Samples for cellular assays were collected in sodium heparin anticoagulant vacutainers. Responses were analyzed as continuous (geometric mean or median) and categorical (positive response or 4-fold increase from baseline), as traditionally reported and defined by Crucell prior to analysis. 

#### Antibody titers against the malaria circumsporozoite antigen

Antibody titers against the malaria circumsporozoite antigen were assessed at days 0, 28, 56, 84, 112 and 140. ELISA was used to determine IgG antibody concentrations to CS protein using a (NANP)_6_ peptide as capture antigen at Crucell as previously described[25]. Briefly, the peptide was obtained from Pepscan (Lelystad, Netherlands) at a purity of 90%. The peptide was coated at a concentration of 2 ug/ml in 0.05M carbonate buffer at 2-8°C overnight or for a maximum of 3 days. Reference sera, serum samples, and internal controls were diluted in phosphate-buffered saline (PBS), 2% gelatin, and 1% human serum (dilution buffer) and incubated for 1 hour at room temperature. Caprine anti-human IgG horseradish peroxidase was added and incubated for 1 h at room temperature. Finally, o-phenylenediamine (OPD; Sigma Aldrich) was added for the colorimetric reaction, which was stopped after 10 min using 5.3% H_2_SO_4_ stop solution. Subsequently, the optical density (OD) was measured at 492 nm, using a Bio-Tek microplate spectrophotometer PowerWave 340. The limit of detection for the anti-CS assay was 18 EU/mL. Values below this threshold were set at 50% of the limit of detection.

#### Neutralizing antibody titers against Adenovirus type 35

The neutralizing antibody titers against Adenovirus type 35 were measured using Adenovirus Neutralization Assay at days 0, 28, 56, 84, 112 and 140. Ad35-specific neutralizing antibody titers were assessed by luciferase-based virus neutralization assays as described previously[26]. The adenovirus neutralization assay has been optimized and validated for human serum. In short, serum was heat inactivated and serially diluted by 2-fold (starting dilution is 1:16). Ad35.Luc solution (10^8^ vp/ml) was added to each well at 500 virus particles per cell. A549 cells were added at 10^4^ cells/well and plates were incubated at 37°C/10% CO_2_ for 24 to 26 hours. After incubation, medium was discarded, PBS was added, and plates ere stored frozen overnight. Plates were allowed to thaw at room temperature, Luciferase Steady-Lite substrate as added and the lysate was transferred into black/white isoplates. Luminescence counts were recorded on a 1450 MicroBeta Trilux. Titers were determined by validated software ANAM (Adenovirus Neutralization Assay Macro). The limit of detection for the Ad35 neutralizing antibody assay was 16 IC_90_. Values below this threshold were set at 50% of the limit of detection.

#### T cell responses against the malaria circumsporozoite antigen

T cell responses against the malaria circumsporozoite antigen were measured by Elispot at days 0, 28, 56, 84, 112 and 140. Thawed and rested PBMC samples were stimulated with a single pool of 59 CS peptides (2μg/ml/peptide), consisting of 15-mer peptides overlapping by 11 amino acids, representing the CS protein. For mock stimulated wells, DMSO was used (0.24%). 

For ELISpot, pre-coated human α-IFNγ ELISpot plates (MabTech, Sweden) were blocked and PBMCs were added to the blocked plates at a concentration of 2x10^5^ viable cells/well for peptide and mock stimulated wells. After overnight incubation the plates were washed and the detection antibody 7-B6-1-ALP (MabTech, Sweden) was added. After washing, the spots were visualized with BCIP/NBT (MabTech, Sweden) and counted using a digital imager and automated spot counting (AID ELISpot reader, AutoImmun-Diagnostika). In each assay an internal control was included to determine whether the assay was valid.

For Intracellular Cytokine Staining (ICS), one million cells per well were stimulated with either CMV or CS peptide pool diluted in medium. Golgi Plug (BD Bioscience, Mountain View, USA) was included during the 6-hour incubation together with peptide stimulation. For live/dead staining, aqua fluorescent Dye A (Invitrogen, the Netherlands) was used for 30 minutes at RT. Staining for 20 minutes at RT to indentify T-cells was performed using APC H7-α-CD3, Horizon V450-α-CD4 and PerCP-Cy5.5-α-CD8 (BD Biosciences, Mountain View, USA) Cells were permeabilized and fixed by Cytofix/cytoperm (BD Biosciences) and thereafter stained for intracellular cytokines using APC-α-IFNγ, FITC-α-TNFα and PE-α-IL-2 (BD Biosciences). Data was acquired and analyzed by FACSCanto II flowcytometer and FACSDiva 6.1.2 software (BD Biosciences). In each assay an internal control was included to determine if the assay was valid. Prior to analyzing samples with FACS Canto II, set-up tracking beads (BD set-up beads, BD Bioscience) and compensation beads (Compbeads, BD Bioscience) were run. Prequalified gating was used throughout the sample analysis.

### Sample size

The sample size of ten per group was proposed to receive the Ad35.CS.01 vaccine to obtain preliminary safety information on a small cohort of subjects before proceeding to subsequent larger trials. The study had 80% power to observe at least one adverse event associated with the vaccine if the true event rate were 10%; however the study was not powered to formally compare dosage-related immune responses.

### Statistical analysis

The study was designed to estimate adverse event rates and immune responses among ascending vaccine dosages rather than to formally test for differences among study groups. Safety and immunogenicity outcomes are reported as frequencies or means with 95% confidence intervals. Fisher’s exact tests for categorical and Kruskal-Wallis tests for continuous variables were used to compare responses in vaccine groups relative to placebo. 

### Ethics statement

Prior to enrollment of subjects into this trial, the protocol and the informed consent form were reviewed and approved by the National Ethical Committee of Burkina Faso. The study was conducted in full conformity with the Declaration of Helsinki, the ICH GCP regulations and guidelines and regulatory requirements of Burkina Faso. The assent of the community was obtained through a series of meetings with community opinion leaders and senior members. Individual written informed consent was obtained from all participants in the presence of an impartial witness for illiterate participants. A qualified and experienced physician not associated with this protocol served as the Independent Safety Monitor. The SMC and the local safety monitor reviewed the cumulative safety data and provided the investigators, through the sponsor, with written authorization to proceed to the next dose escalation at each stage. 

## Results

### Population characterization

The study was conducted from April 2010 to December 2011. Subjects were followed for approximately 12 months. One hundred thirty-seven adults consented to screening; of these, sixty-one volunteers were eligible and 48 (21 females and 27 males) were enrolled. Eight subjects were randomized to receive placebo and 40 to receive one of four ascending dosages of Ad35.CS.01. 

**Figure 1:**
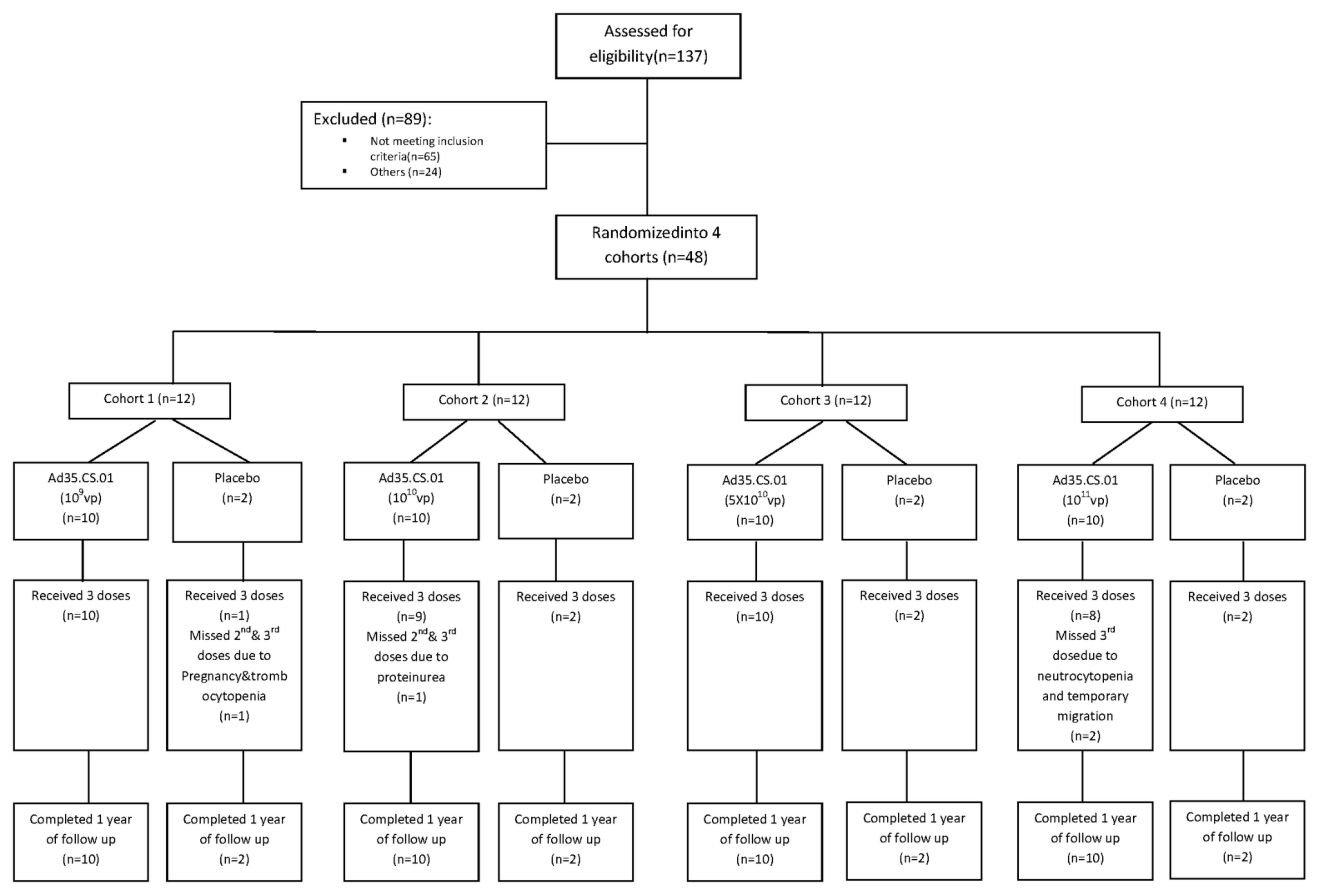
Trial profile.

All enrolled participants received at least one vaccine dose and completed one year follow up for ascertainment of adverse events. All ten subjects in the first (Ad35.CS.01 10^9^ vp) and third (Ad35.CS.01 5X10^10^ vp) dose groups received all three vaccinations. Four subjects in the remaining study groups did not receive all three vaccinations (Figure 1); one subject each in the second (Ad35.CS.01 10^10^ vp) fourth (Ad35.CS.01 10^11^ vp) dose and placebo groups were withdrawn by investigator due to adverse events after the first or second vaccination (proteinuria, neutrocytopenia, and thrombocytopenia, respectively). One subject in the fourth dose group did not receive the third vaccination due to migration from the study area. Study groups were comparable in baseline demography, height, weight, vital signs, and laboratory assessment (Table 1). 

**Table 1:** Demographic, Clinical, and Laboratory Baseline Characteristics of enrolled subjects.

**Characteristic**	**10^9^ vp/mL**	**10^10^ vp/mL**	**5x10^10^ vp/mL**	**10^11^ vp/mL**	**Placebo**
Age (years)	31.9 (7.3)	25.2 (6.8)	28.8 (10.9)	31.7 (7.7)	33.5 (9.7)
Women (%)	40.0	40.0	50.0	50.0	37.5
Height (cm)	168.8 (7.2)	164.6 (8.2)	168.5 (7.7)	165.3 (6.5)	165.8 (13.5)
Weight (Kg)	59.2 (9.8)	59.6 (6.7)	59.8 (3.7)	58.5 (6.4)	60.8 (13.0)
WBC (10^3^ cells /µL)	7.2 (1.05)	7.0 (1.13)	5.6 (0.90)	4.7 (0.71)	6.0 (1.32)
Hemoglobin (g/dL)	14.2 (1.25)	14.9 (1.23)	13.7 (1.27)	13.9 (1.48)	14.5 (1.53)
Platelets (10^3^ cells /µL)	261 (40.7)	295 (60.7)	280 (45.7)	257 (66.7)	222 (64.3)
ALT (U/L)	17 (14.0)	19 (6.3)	21 (9.1)	21 (8.1)	22 (8.9)
AST (U/L)	24 (9.0)	26 (6.3)	26 (6.0)	29 (11.2)	27 (9.5)
Creatinine (mg/dL)	0.9 (0.10)	0.9 (0.09)	0.9 (0.11)	0.8 (0.11)	0.9 (0.10)
Glucose (mg/dL)	87 (8.3)	87 (2.8)	89 (7.2)	82 (7.6)	90 (9.4)
Sodium (mmol/L)	146 (1.8)	144 (1.7)	145 (1.4)	144 (1.2)	145 (1.2)
Potassium (mmol/L)	4.3 (0.17)	4.5 (0.27)	4.5 (0.14)	3.9 (0.21)	4.4 (0.43)

Mean (SD) shown for continuous variables.

### Safety and reactogenicity

#### Local Solicited Reactogenicities

Thirteen (27.1%) subjects experienced local symptoms consisting of erythema, induration, and pain or tenderness (Table 2). No local reactions occurred in the placebo group. Frequency of local symptoms was not associated to vaccine dosage. All local reactions were mild and defined as vaccine related. Both the test article and the placebo were well-tolerated.

**Table 2:** Local and systemic solicited reactogenicities over 14 day follow up by treatment group.

	**10^9^ vp/mL**	**10^10^ vp/mL**	**5x10^10^ vp/mL**	**10^11^ vp/mL**	**Placebo**
**Solicited symptom**	**N (%)**	**N (%)**	**N (%)**	**N (%)**	**N (%)**
Elevated axillary temperature	0 ( 0.0)	1 (10.0)	0 ( 0.0)	0 ( 0.0)	0 ( 0.0)
Headache	5 (50.0)	5 (50.0)	5 (50.0)	3 (30.0)	2 (25.0)
Malaise	0 ( 0.0)	0 ( 0.0)	0 ( 0.0)	0 ( 0.0)	0 ( 0.0)
Myalgia	0 ( 0.0)	0 ( 0.0)	0 ( 0.0)	1 (10.0)	0 ( 0.0)
Chills	0 ( 0.0)	1 (10.0)	0 ( 0.0)	0 ( 0.0)	0 ( 0.0)
Nausea	0 ( 0.0)	1 (10.0)	1 (10.0)	0 ( 0.0)	0 ( 0.0)
Vomiting	0 ( 0.0)	1 (10.0)	0 ( 0.0)	0 ( 0.0)	1 (12.5)
Maximum systemic symptoms	5 (50.0)	7 (70.0)	5 (50.0)	4 (40.0)	3 (37.5)
Pain	1 (10.0)	1 (10.0)	4 (40.0)	5 (50.0)	0 ( 0.0)
Induration	2 (20.0)	0 ( 0.0)	1 (10.0)	0 ( 0.0)	0 ( 0.0)
Erythema	1 (10.0)	0 ( 0.0)	0 ( 0.0)	0 ( 0.0)	0 ( 0.0)
Ecchymosis	0 ( 0.0)	0 ( 0.0)	0 ( 0.0)	0 ( 0.0)	0 ( 0.0)
Maximum local symptoms	3 (30.0)	1 (10.0)	4 (40.0)	5 (50.0)	0 ( 0.0)
Any symptoms	6 (60.0)	7 (70.0)	5 (50.0)	7 (70.0)	3 (37.5)

#### Systemic Solicited Reactogenicities

 Sixteen (33.3%) subjects experienced mild and 7 (14.6%) moderate systemic symptoms (Table 2). Mild symptoms consisted of myalgia, chills, headache, nausea, fever and vomiting and all moderate events were headache. No grade 3 solicited symptoms were observed. Frequency of symptoms was similar among study and placebo groups.

#### Unsolicited Adverse Events

Unsolicited systemic reactions/events were ascertained at each clinic visit from day 0 to day 112. Of 165 non-serious adverse events, 17 mild and 1 moderate events were evaluated as associated to study vaccine. Associated adverse events were most frequently classified as mild abnormal laboratory values occurring within 7 days of vaccination. Approximately 70% of subjects in each study group experienced at least one infection/infestation over follow-up; events of this class were commonly attributed to upper respiratory tract infections and malaria. One serious adverse event not related to the study vaccine occurred in the Ad35.CS.01 10^10^ vp/mL vaccine group. The event was described as liver abscess due to amoeba attributed to poor food hygiene. The event resolved without sequellae. One pregnancy occurred in the placebo group and resulted in a miscarriage.

Other commonly reported classes of events, occurring in approximately 25% of subjects in each study group, were nervous system disorders dominated by headache and gastro-intestinal disorders by abdominal pain. All adverse events resolved without sequellae. Frequency of events was similar among study and placebo groups. 

#### Laboratory safety tests

Mild and moderate abnormalities frequently included hematuria, elevated glucose, and low or high white blood cell count. Severe abnormalities consisted of hematuria, attributed to menstruation (N = 3) or urinary tract infection (N = 1), and elevated glucose (N = 1) identified by investigator as not clinically significant because the subject hadn’t been fasting at the time of blood draw. Upon retesting the following day, the blood glucose was found to be within the normal limits. 

Ten percent or more of subjects in each treatment group and placebo presented with hematuria, considered not associated to vaccine where attributed to menstruation or urinary tract infection. Proteinuria and neutropenia in the Ad35.CS.01 10^10,^ Ad35.CS.01 5x10^10^ or Ad35.CS.01 10^11^ dose groups were evaluated as associated to vaccine where an alternate etiology was not identified. Thrombocytopenia occurred in one subject in the placebo group. 

### Immunogenicity: Humoral Immune Response

Secondary outcomes of this study were IgG antibody responses against the circumsporozoite antigen and neutralizing antibody responses against the vaccine vector Adenovirus type 35. The geometric mean of anti-CS and anti-Ad35 neutralizing antibody responses were evaluated at day 0, 28, 56, 84, 112, and 140.

#### CS Antibody Response

**Figure 2:**
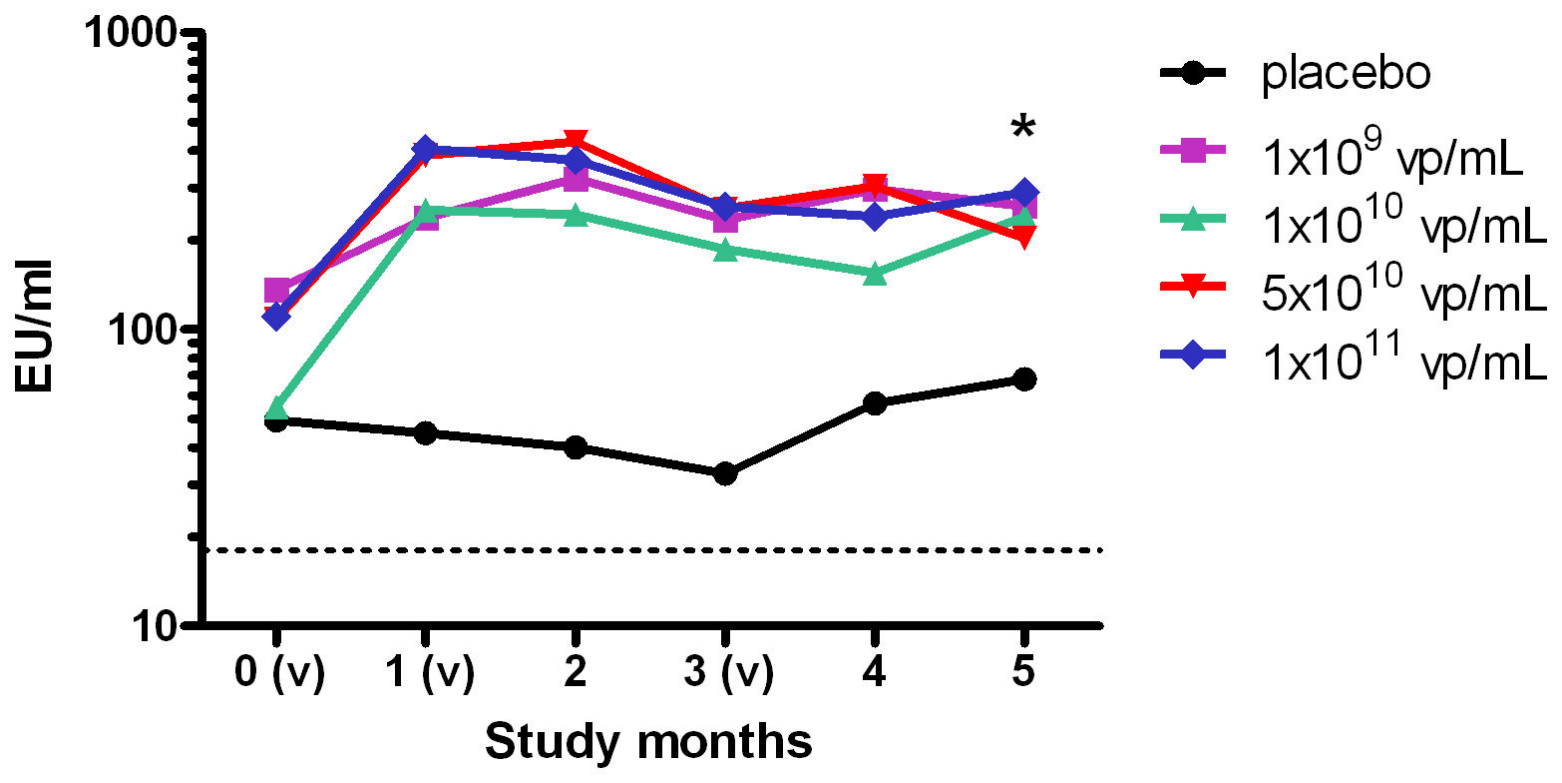
Geometric Mean Anti-CS Antibody Response Over Time. Geomean anti-CS antibody titers are plotted per timepoint at baseline, 1 month after the first vaccination, 1 month after the second vaccination, 2 months after the second vaccination and at 1 and 2 months after the third vaccination. All dose groups results are shown (nr of samples assessed for any time point): placebo (7 to 8), 10^9^ vp/ml (10), 10^10^ vp/mL (9 to 10), 5x10^10^ vp/mL (10) and 10^11^ vp/mL (7 to 10). (v) designates vaccination on the study timescale, at month 0, 1 and 3. Krusal-Wallis tests were used to compare responses relative to placebo with * indicating p<0.01. EU are relative ELISA Units. The LLOQ is 18 EU/mL (dotted line).

At day 0, the geometric means of baseline antibody responses with specificity to CS were found elevated with similar titers among vaccine and placebo groups, reflecting a maturity of naturally acquired anti-CS immune responses in the study population. Anti-CS antibody levels were markedly raised following product administration among vaccine recipients only (p < 0.01 for difference in median change from baseline to day 140 among vaccine groups relative to placebo) (Figure 2). This effect was most evident after the first vaccine administration.

Four-fold or greater increase from baseline in anti-CS antibody response was most frequent at day 56, occurring in 56%, 70% and 20% of subjects who received the 10^10^, 5 x 10^10^ vp/mL, and 10^11^ vp/mL Ad35.CS.01 doses respectively. Four-fold increases in anti-CS antibody response over this period were not evident in the 10^9^ or placebo groups. 

Level of CS antibody response did not increase substantially from second and third administrations of Ad35.CS.01 from day 28 to day 140 within vaccine groups (Figure 2). Geometric mean antibody titers on Day 140 were higher in the Ad35.CS.01 5x10^10^ and 10^11^ groups relative to other study groups, however statistically significant only relative to placebo: geometric mean (95% CI) (EU/mL) was 205 (124, 341) and 256 (117, 558) in these ascending dosage groups versus 74 (34, 162) in the placebo group; p = 0.03 and p=0.02 respectively.

#### Ad35 Neutralizing Antibody Response

**Figure 3:**
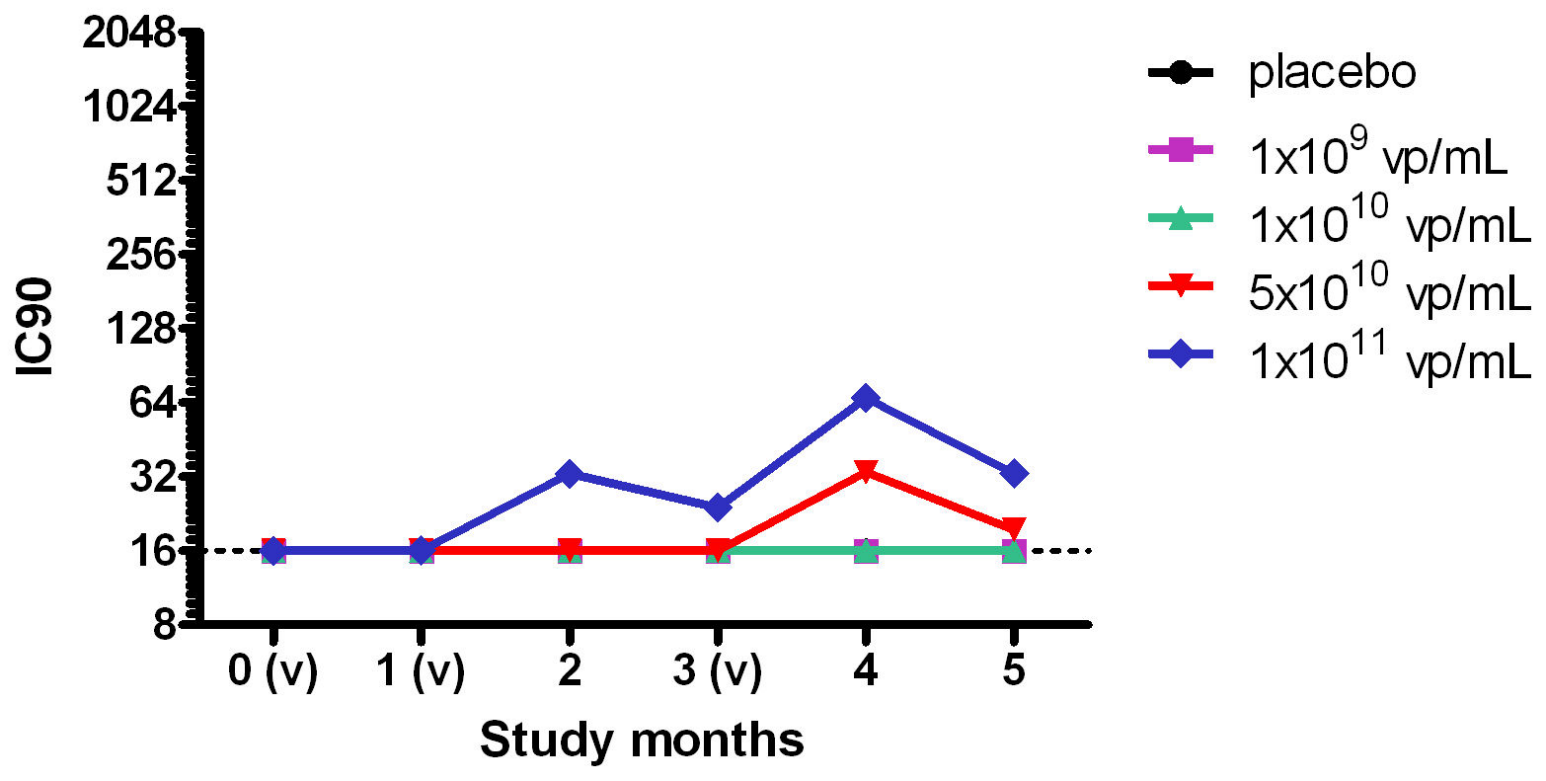
Geometric Mean Anti-Ad35 Neutralizing Antibody Response Over Time. Geomean Ad35 neutralizing antibody titers are plotted per timepoint at baseline, 1 month after the first vaccination, 1 month after the second vaccination, 2 months after the second vaccination and at 1 and 2 months after the third vaccination. All dose groups results are shown (nr of samples assessed for any time point): placebo (7 to 8), 10^9^ vp/ml (10), 10^10^ vp/mL (9 to 10), 5x10^10^ vp/mL (10) and 10^11^ vp/mL (8 to 10). (v) designates vaccination on the study timescale, at month 0, 1 and 3. IC90 designates the 90% inhibitory concentration. 16 is the LLOQ (dotted line).

Baseline geometric mean anti-Ad35 neutralizing antibody response was similar among study groups in the 5 groups (range 10-14 IC90). Frequency of subjects with positive response (IC90 > 16) at baseline ranged from 10-30% which is similar to seroprevalence data published previously[17]. The rate of positive responses increased from baseline to post-vaccination in vaccine groups only. In all dose groups seropositive titers were detected upon vaccine administrations, however, some subjects showed negative Ad35 neutralizing antibody levels throughout the study. Among vaccine groups, peak titers of Ad35 neutralizing antibodies were observed one month after the third vaccination. Following 3 administrations of the Ad35 vaccine, levels of neutralizing antibodies, however, remained modest with low to medium titers. The development of Ad35 neutralizing antibodies was dose dependent, with higher numbers of seropositives and higher titers in the higher dose groups (Figure 3). 

### Immunogenicity: Cellular Immune Response

Blood was drawn for assessment of cellular immune response at six visits: day 0 (prior to the first vaccination); day 28 (prior to the second vaccination); day 56 (28 days after the second vaccination); day 84 (prior to the third vaccination); day 112 (28 days after the third vaccination); and day 140 (56 days after the third vaccination).

#### Cell-mediated responses

**Figure 4:**
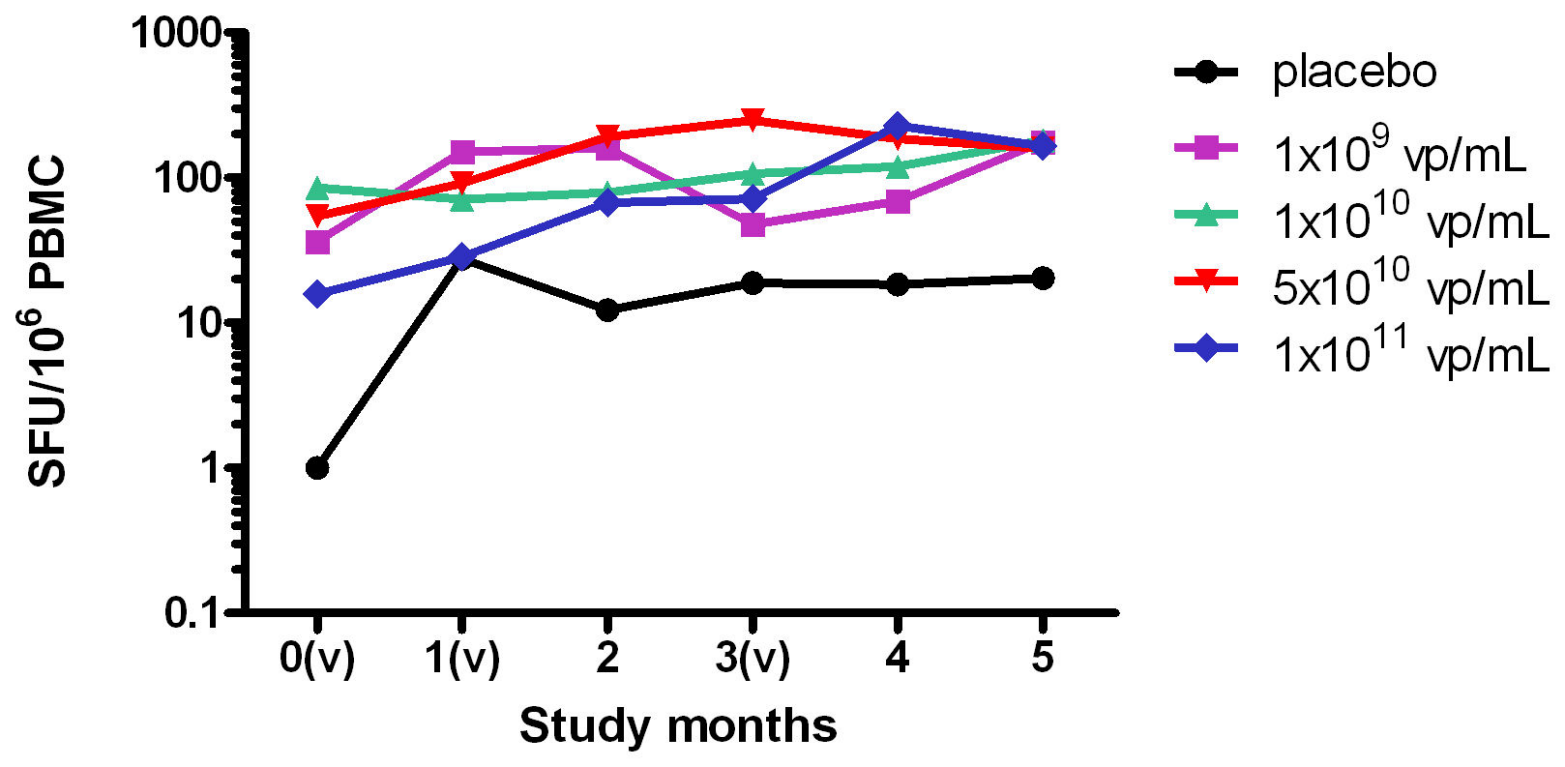
Median Elispot T Cell Response. Median mock subtracted IFNγ Elispot responses are plotted per timepoint at baseline, 1 month after the first vaccination, 1 month after the second vaccination, 2 months after the second vaccination and at 1 and 2 months after the third vaccination. All dose groups results are shown (nr of samples assessed for any time point): placebo (5 to 7), 10^9^ vp/ml (8 to 10), 10^10^ vp/mL (9 to 10), 5x10^10^ vp/mL (8 to 10) and 10^11^ vp/mL (7 to 10). (v) designates vaccination on the study timescale, at month 0, 1 and 3. Krusal-Wallis tests were used to compare responses relative to placebo. SFU means Spot forming Units.

Baseline Elispot responses were not statistically different among vaccine groups relative to placebo. Median response increased after first vaccination in all study groups however remained lowest in placebo group throughout follow-up (Figure 4). Median (range) change in response from Day 0 to Day 140 was 85 (-929-1287) SFU/million among vaccine groups vs. 15 (0-21) SFU/million among placebo (p = 0.17). 

**Figure 5:**
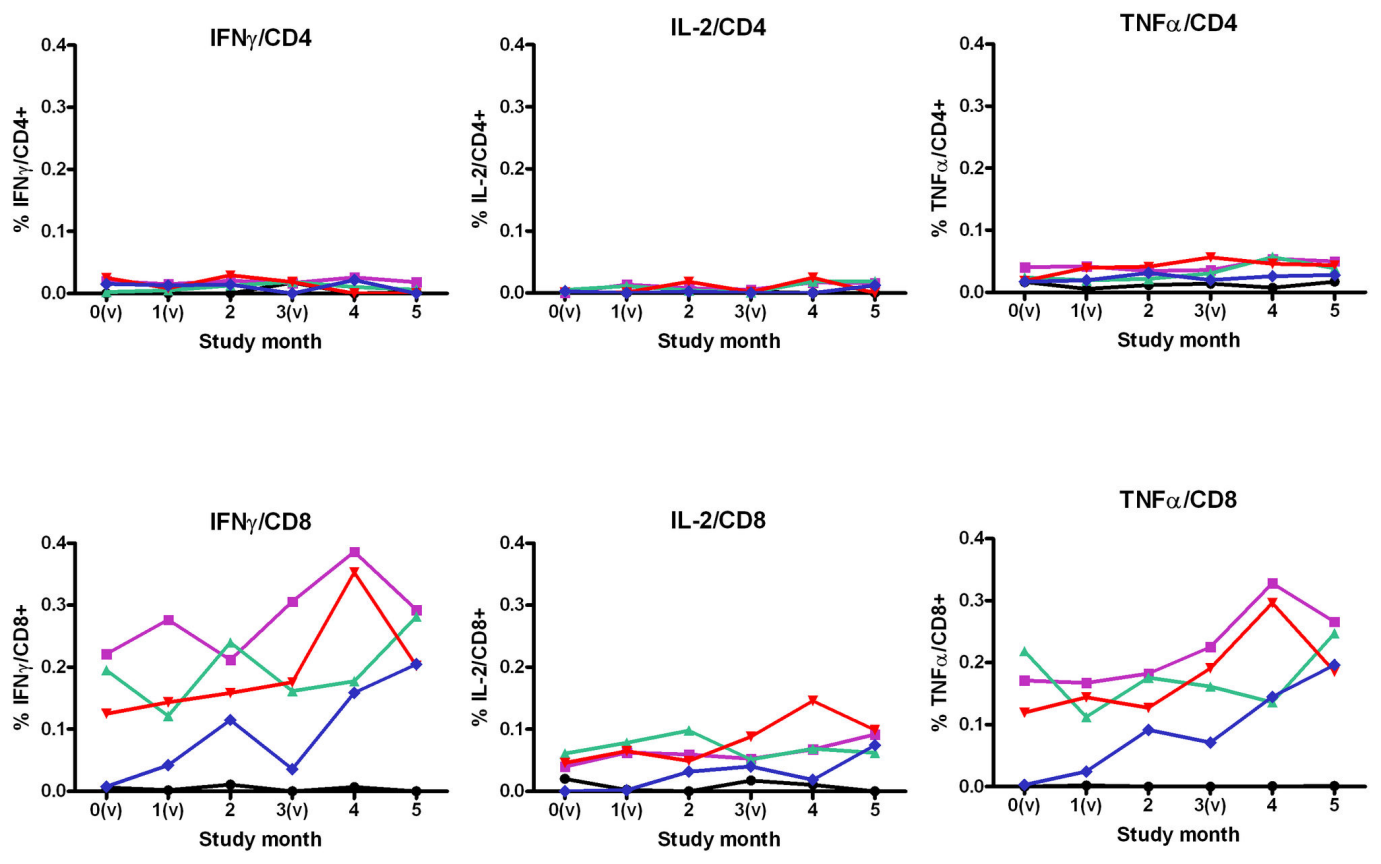
Median ICS T Cell Response. Median mock subtracted ICS responses are plotted per timepoint at baseline, 1 month after the first vaccination, 1 month after the second vaccination, 2 months after the second vaccination and at 1 and 2 months after the third vaccination. All dose groups results are shown (nr of samples assessed for any time point): placebo (6 to 8), 10^9^ vp/ml (10), 10^10^ vp/mL (9 to 10), 5x10^10^ vp/mL (7 to 9) and 10^11^ vp/mL (7 to 8). (v) designates vaccination on the study timescale, at month 0, 1 and 3..

CD4 T cell responses as determined by Intracellular Cytokine Staining (ICS) remained low for the three cytokines assays, IFNγ, TNFα and IL-2 throughout the study for all study groups (Figure 5). 

Median CD8 T-cell response increased approximately two-fold among vaccine groups from baseline to one month after the third vaccination. CD8 T cells produced mainly IFNγ and TNFα, with very little IL-2, with median responses reaching maximally 0.4% cytokine responses within CD8 T cells. Placebo group responses remained near 0% for the duration of the study (Figure 5).

## Discussion

We evaluated the reactogenicity, safety and immunogenicity of four ascending dosages of Ad35.CS.01 malaria candidate vaccine, administered to 48 adult volunteers living in an area endemic for *P. falciparum* malaria in Burkina Faso. An ascending dosage design was selected because this vaccine had not previously been administered in a malaria- experienced population. The four dosages of Ad35.CS.01 vaccine were safe and modestly immunogenic. 

No subjects experienced a grade 3 solicited reaction. Frequency of systemic reactions was similar among vaccine and placebo groups and did not appear dose-dependent. The most common local symptoms were mild or moderate pain or induration. All symptoms at the site of injection resolved spontaneously within 14 days of vaccination. 

 Frequency of non-serious events was similar among study groups and most commonly consisted of headache and vomiting (solicited adverse events) and mild or moderate gastrointestinal disorders or infections/infestations (unsolicited adverse events). Associated events were most frequently classified as mild abnormal laboratory values occurring within 7 days post-vaccination and included hematuria, proteinuria and leucocytopenia. 

There were similar incidences and intensity of general and unsolicited symptoms among subjects receiving Ad35.CS.01 and those receiving the placebo. The number of volunteers who had biological values outside the normal ranges for the measured parameters was similar in the Ad35.CS.01 and placebo groups. It should be noted that abnormal values were defined according to international normal ranges which may not be appropriate to our population. Thrombocytopenia and proteinuria events that resulted in discontinuation of vaccination of two subjects were found resolved at final follow up. 

Effective immune protection against clinical malaria involves the acquisition of cellular and humoral immunity, which may naturally occur after repeated exposure to malaria or develop upon anti-malarial vaccination. Antibodies to the circumsporozoite protein have been reported to play a key role in protection against malaria in a number of epidemiological studies[27-32]. In this trial, the Ad35.CS.01 vaccine induced increased levels of malaria humoral immune responses among vaccine groups. The effect did not substantially vary among vaccine dosage groups. Prevalence and level of neutralizing antibodies against Ad35 also increased post immunization, although the geometric mean response remained low among all study groups (IC90 range 10 to 68). These data are consistent with reports that neutralizing antibodies against Ad35 are rare in humans worldwide or present in low titers [17,33-35]. Previous studies have also described the ability of Ad35 vectors to deliver Plasmodium antigens and elicit immunogenicity as well as protect laboratory animals from sporozoite challenge with *P yoelii*[36-38]. Results of the current study build on the more recently demonstrated induction of antibody response by a prime-boost regimen including Ad35.CS in rhesus macaques[39,40]; this vaccination approach is hypothesized to elicit a more complex and advantageous immune response relative to homologous boosting.

Adenoviral vectors offer the potential to safely induce CD4 and CD8 T-Cells in animals[36,40] and humans[19,20] . IFNγ producing CD8 and CD4 T-cells may be required for clearance of parasite-infected hepacytes[41]. In the current study immunization with Ad35.CS.01 induced sustained responses of IFNγ and TNFα producing CD8 T cells. A similar response was not seen with CS specific CD4 T cells. Induction of IFNγ producing CD8 T-cells by adenovirus vectors has previously been described in both mice and macaques[39]. Our results are consistent with the acceptable safety of the Ad35.CS.01 vaccine demonstrated in malaria naïve adults living in the US (manuscript submitted). The similar results profile in the current semi-immune population is expected to generalize to adults living in malaria endemic areas. It has recently been shown that Ad35 vectors expressing TB antigens also expressed in BCG induce more robust responses in adults previously primed with BCG then in BCG naïve individuals suggesting that Ad35 vectors may be more potent in humans as boosters then as priming vaccines [42]. Despite exposure to malaria sporozoites in this malaria experienced population, priming for cellular immune responses may not occur as suggested by the low baseline Elispot responses in most individuals. It would therefore be useful to explore this Ad35 vectored CS vaccine in prime boost regimens with other adjuvants to enhance both antibody and cellular responses.

The acceptable safety and tolerability and modest immunogenicity of Ad35.CS.01 in the malaria semi-immune adult population described here, positions this vaccine for future evaluation of CS specific antibody and cellular response induction. Alternative strategies that may be considered are homologous or heterologous prime boost regimens with varied CS protein formulations. Success of such regimens may demonstrate a value for future efficacy studies in human challenge models.

## Supporting Information

Checklist S1 **CONSORT Checklist.**
(DOC)

Protocol S1 **Trial Protocol.**
(PDF)
