# Supplementary material for: A Phase 1b Randomized, Controlled, Double-Blinded Dosage-Escalation Trial to Evaluate the Safety, Reactogenicity and Immunogenicity of an Adenovirus Type 35 Based Circumsporozoite Malaria Vaccine in Burkinabe Healthy Adults 18 to 45 Years of Age
Source: PLoS One. 2013 Nov 11;8(11):e78679. doi: 10.1371/journal.pone.0078679 (PMC3823848; doi:10.1371/journal.pone.0078679)
Supplement: Protocol S1 — Trial Protocol. (PDF) [file pone.0078679.s002.pdf]

A PHASE 1 RANDOMIZED, CONTROLLED, DOUBLE-BLINDED DOSAGE-ESCALATION TRIAL TO EVALUATE THE IMMUNOGENICITY, SAFETY, AND REACTOGENICITY OF AN ADENOVIRUS TYPE 35 BASED CIRCUMSPOROZOITE MALARIA VACCINE IN BURKINABE SEMI-IMMUNE, HEALTHY ADULTS 18 TO 45 YEARS OF AGE

DMID Protocol Number: 08-0037

DMID Funding Mechanism: Contract Support 400016C

IND Sponsor: DMID

Principal Investigator: Sodiomon Bienvenu Sirima, MD, PhD

DMID Protocol Champion: Steven Rosenthal, MD, MPH

DMID Clinical Protocol Manager: Walter Jones, RN, MPH

DMID Medical Monitor: Georgia Latham, MD

DMID Regulatory Affairs Specialist: Blossom Smith, MS

Version Number 4.0

August 31, 2011

STATEMENT OF COMPLIANCE

This trial will be conducted in compliance with the protocol, International Conference on Harmonisation (ICH) Topic E6: Guideline for Good Clinical Practice (GCP), and the applicable regulatory requirements from United States (US) Code of Federal Regulations (CFR) (Title 45 CFR Part 46 and Title 21 CFR including Parts 50, 56 and 312) concerning informed consent, Institutional Review Board (IRB) and Investigational New Drug Application (IND) regulations.

SIGNATURE PAGE

The signature below constitutes the approval of this protocol and the attachments, and provides the necessary assurances that this trial will be conducted according to all stipulations of the protocol, including all statements regarding confidentiality, and according to local legal and regulatory requirements and applicable US federal regulations and ICH guidelines.

Site Investigator:

Signed: ______________________________ Date: ______________

Sodiomon B. Sirima, MD, PhD

______________________________ ______________

TABLE OF CONTENTS

Page
- Abbreviations vii
- Protocol Summary viii

1 Key Roles 1
2 Background Information and Scientific Rationale 4
2.1 Background Information 4
2.1.1 Pre-Clinical and Toxicology Studies 5
2.1.2 Clinical Experience 6
2.2 Rationale 7
2.3 Potential Risks and Benefits 7
2.3.1 Potential Risks 7
2.3.2 Known Potential Benefits 8
3 Objectives 9
3.1 Study Objectives 9
3.2 Study Outcome Measures 9
Primary Outcome Measure 9
3.2.1 9
 The number of subjects experiencing severe (Grade 3) injection site reactions within 14 days following vaccination. 9
 The number of subjects experiencing severe (Grade 3) systemic reactions within 14 days following vaccination. 9
 The number of subjects with severe or life-threatening adverse events considered associated with vaccination at any point during the study period. 9
3.2.2 Secondary Outcome Measures 9
4 Study Design 11
5 Study POPULATION 12
5.1 Provision of informed consent before any protocol procedures are performed. 13
5.2 Enrollment Within Each Dosage Cohort 12
5.3 Subject Inclusion Criteria 13
5.4 Subject Exclusion Criteria 14
5.5 Treatment Assignment Procedures 15
5.5.1 Randomization Procedures 15
5.5.2 Blinding Procedures 15
5.5.3 Reasons for Withdrawal from Vaccination Schedule 16
5.5.4 Handling of Subjects Who Discontinue Vaccination 16
6 Study Intervention/Investigational Product 17
6.1 Study Product Description 17
6.1.1 Acquisition 17
6.1.2 Formulation, Packaging, and Labeling 17

6.1.3 Product Storage and Stability 17
6.2 Dosage, Preparation and Administration of Investigational Product 18
6.3 Modification of Investigational Product for a Participant 18
6.4 Accountability Procedures for the Study Investigational Products 18
6.5 Concomitant Medications/Treatments 18
7 Study Schedule 19
7.1 Screening Day -42 to -1, Visit 1 19
7.2 Enrollment/Baseline Day 0, Visit 2 19
7.3 Follow-up 20
7.3.1 Day 1 (+1), 2A, Scheduled home visit 20
7.3.2 Day 2 (+1), Visit 3 21
7.3.3 Day 7 (+ 2), Visit 4 22
7.3.4 Day 14 (+ 3), Visit 5 22
7.3.5 Day 28 (window period: -3 days to +3 days), Visit 6, 2nd vaccination 22
7.3.6 Day 1 (+1) post-2nd vaccination, Visit 6A, Scheduled home visit 23
7.3.7 Day 2 (+1) post-2nd vaccination, Visit 7 24
7.3.8 Day 7 (+2) post-2nd vaccination, Visit 8 24
7.3.9 Day 14 (+3) post-2nd vaccination, Visit 9 25
7.3.10 Day 28 (+3) post-2nd vaccination, Visit 10, 3rd vaccination 25
7.3.11 Day 1(+1) post-3rd vaccination, Visit 10 A, Scheduled home visit 26
7.3.12 Day 7 (+2) post-3rd vaccination, Visit 12 27
7.3.13 Day 14 (+3) post-3rd vaccination, Visit 13 27
7.3.14 Day 28 (+3) post-3rd vaccination, Visit 14 27
7.3.15 Day 112 (+1 month), Visit 15 28
7.3.16 Day 172 (+1 month) Visit 16 28
7.3.17 Day 232 (+1 month) Visit 17 28
7.3.18 Day 292 (+1 month) Visit 18 29
7.3.19 Day 365 (+1 month) Visit 19 29
7.4 Early Termination 29
7.5 Early Termination Visit 30
7.6 Unscheduled Visit 30
8 Study Procedures/Evaluations 31
8.1 Clinical Evaluations 31
8.2 Laboratory Evaluations 31
8.2.1 Clinical Laboratory Evaluations 31
8.2.2 Humoral and Cell-mediated Immune Assays 32
8.2.3 Specimen Preparation, Handling, and Shipping 33
9 Assessment of Safety 35
9.1 Methods and Timing for Assessing, Recording, and Analyzing Safety Parameters 35
9.1.1 Adverse Events, Reactogenicity, Serious Adverse Events 35
9.2 Reporting Procedures 38

9.2.1 Serious Adverse Event Detection and Reporting........38
9.2.2 Regulatory Reporting for Studies Conducted Under DMID-Sponsored Investigational New Drug (IND) Application........39
9.2.3 Reporting of Pregnancy........40
9.2.4 Type and Duration of the Follow-up of Subjects after Adverse Events........40
9.3 Halting Rules........40
9.4 Safety Oversight (Independent Safety Monitor plus SMC)........41
10 Clinical Monitoring........43
10.1 Site Monitoring Plan........43
11 Statistical Considerations........44
11.1 Study Overview........44
11.2 Final Analysis Plan........45
12 Source Documents and Access to Source Data/Documents........46
13 Quality Control and Quality Assurance........47
14 Ethics/Protection of Human Subjects........48
14.1 Declaration of Helsinki........48
14.2 Institutional Review Board (IRB)........48
14.3 Informed Consent Process........48
14.4 Exclusion of Women, Minorities, and Children (Special Populations)........49
14.5 Subject Confidentiality........49
14.6 Study Discontinuation........50
15 Data Handling and Record Keeping........51
15.1 Data Management Responsibilities........51
15.2 Data Capture Methods........51
15.3 Types of Data........52
15.4 Timing/Reports........52
15.5 Study Records Retention........52
15.6 Protocol Deviations........52
16 Publication Policy........54
17 Literature References........55
Appendix A: Schedule of Events 56
Appendix B: Laboratory Adverse Event Grading Scale[8]........58

ABBREVIATIONS

Ab	Antibodies
Ad	Adenovirus

AdvantageEDC^{SM}	The EMMES Corporation’s web-based data entry system
AE	Adverse Event/Adverse Experience
CFR	Code of Federal Regulations
CMI	Cell Mediated Immunity
CRF	Case Report Form
CS	Circumsporozoite
DCC	Data Coordinating Center
DHHS	Department of Health and Human Services
DMID	Division of Microbiology and Infectious Diseases, NIAID, NIH, DHHS
eCRF	Electronic Case Report Form
ELISA	Enzyme-linked immunosorbent assay
FDA	Food and Drug Administration
GCP	Good Clinical Practice
HIPAA	Health Insurance Portability and Accountability Act
ICH	International Conference on Harmonisation
ICMJE	International Committee of Medical Journal Editors
IEC	Independent or Institutional Ethics Committee
IND	Investigational New Drug Application
IRB	Institutional Review Board
ISM	Independent Safety Monitor
MedDRA ®	Medical Dictionary for Regulatory Activities
MOP	Manual of Procedures
NIAID	National Institute of Allergy and Infectious Diseases, NIH, DHHS
NIH	National Institutes of Health
PI	Principal Investigator
PE	Physical Examination
PBMC	Peripheral Blood Mononuclear Cells
SAE	Serious Adverse Event/Serious Adverse Experience
SMC	Safety Monitoring Committee
US	United States
vp	Viral particles

PROTOCOL SUMMARY

Title: A Phase 1 Randomized, Controlled, Double-Blinded, Dosage-Escalation Trial to Evaluate the Immunogenicity, Safety, and Reactogenicity of an Adenovirus Type 35 Based Circumsporozoite Malaria Vaccine in Burkinabè, Semi-Immune, Healthy Adults 18 to 45 Years of Age.

Phase: I

Population: 48 healthy male and female subjects aged 18 to 45 years in Burkina Faso.

Site: Projet de Développement de Vaccins Anti-Paludique- Centre National de Recherche et de Formation sur le Paludisme (PDVAP/CNRFP) Burkina Faso

Study Duration: Approximately 12 months per subject .

Description of Agent or Intervention: Adenovirus Type 35 Circumsporozoite Malaria Vaccine (Ad35.CS.01) versus Normal Saline Placebo Control via Intramuscular Injection.

Objectives:

Primary Objectives:

- To assess the safety and reactogenicity of ascending dosages of Adenovirus Type 35 based circumsporozoite malaria vaccine among healthy, semi-immune, subjects given in 3 intramuscular doses at 0, 1 and 3 months.

Secondary Objectives:

- To evaluate the immunogenicity of the Adenovirus Type 35 based circumsporozoite malaria vaccine through performance of Humoral Immune Assays (ELISA [enzyme-linked immunosorbent assay] for antibodies to circumsporozoite antigen and Adenovirus Neutralization Assay for neutralizing antibodies to Adenovirus type 35) and Cellular Immune Assays (Elispot and Flow Cytometry) for CS-specific $CD4^+$ and $CD8^+$ T cell responses and a whole blood ELISA assay to measure secreted cytokines.

Exploratory Objectives:

- To evaluate the immunogenicity of the Adenovirus Type 35 based circumsporozoite malaria vaccine through performance of Cellular Immune Assays (Elispot and Flow

Cytometry) for CS-specific $CD4^+$ and $CD8^+$ T cell responses and a whole blood ELISA assay to measure cytokines

Description of Study Design:

Subjects will receive 3 doses of the vaccine by the intramuscular route at 0, 1 and 3 months. The safety, reactogenicity and immunogenicity of ascending dosages of the vaccine will be assessed. Ten subjects will receive vaccine at each of the following dosage levels: 10^9 viral particles (vp)/mL, 10^{10} vp/mL, 5 x 10^{10} vp/mL and 10^{11}vp/mL. Two subjects in each group will receive normal saline as placebo control in each dose group. Dosage escalation will proceed only after review of the safety data by the SMC of the prior dosage level.

**Group**	**Vaccine Formulation** Ad35.CS.01	**Number of Vaccine/Placebo**
A	10^9 vp/mL	10/2
B	10^{10} vp/mL	10/2
C	5 x 10^{10} vp/mL	10/2
D	10^{11} vp/mL	10/2
Total		40/8

Outcome Measures:

Primary:

- The number of subjects experiencing severe (Grade 3) solicited injection site reactions within 14 days following vaccination.
- The number of subjects experiencing severe (Grade 3) solicited systemic reactions within 14 days following vaccination.
- The number of subjects spontaneously reporting severe (Grade 3) adverse events considered associated with the vaccination at any point during the study period.
- Serious adverse events considered associated with the vaccination reported at any point during the study period.

Secondary:

- Antibody titers against the malaria circumsporozoite antigen at days 0, 28, 56, 84, 112 and 140 (Geometric Mean Titer and individual log ELISA units).

- Neutralizing antibody titers against Adenovirus type 35 by Adenovirus Neutralization Assay (GMT).1

Exploratory:

- T cell responses against the malaria circumsporozoite antigen by Elispot at days 0, 28, 56, 84, 112 and 140 .
- T cell responses against the malaria circumsporozoite antigen by Flow Cytometry at days 0, 28, 56, 84, 112 and 140.
- T cell responses against the malaria circumsporozoite antigen by a whole blood assays to measure secreted cytokines.

1 KEY ROLES

Individuals:

Principal Investigator:
Sodiomon Bienvenu SIRIMA, BA, MD, PhD
PDVAP/CNRFP
1487 Avenue de l'Oubritenga
01 BP 2208 Ouagadougou 01
Burkina Faso
Phone: + 226 50 32 46 95/6
Fax: + 226 50 30 52 20 or + 226 50 31 04 77
Email: s.sirima.cnlp@fasonet.bf

Protocol Champion:
Steven Rosenthal, MD, MPH
NIH/NIAID/DMID/PIPB
6610 Rockledge Drive, Room 5067
Bethesda, MD 20892-6603
Phone: 301-496-2544
Fax: 301-402-0659
Email: rosenthals@niaid.nih.gov

Clinical Protocol Manager:
Walter Jones, RN, MPH
NIH/NIAID/DMID/PIPB
6610 Rockledge Drive, Room 5093
Bethesda, MD 20892-6603
Phone: 301-451-3039
Fax: 301-480-0728
Email: joneswalter@niaid.nih.gov

DMID Medical Monitor
Georgia Latham, MD
DMID/NIAID
Office of Clinical Research Affairs
6610 Rockledge Drive
MSC 6603, Room 4506
Bethesda, MD 20892-6603
Tel: 301-443-7707
Fax: 301-402-0659
Email: lathamgs@mail.nih.gov

Independent Safety Monitors:
Fousséni Dao MD
National University Hospital Yalgado Ouédraogo
03 BP 7022 Ouagadougou 03 Burkina Faso
Tel. (00226) 50 31 16 55 / 56 / 57

Fax: (00226) 50 31 18 48.

Institutions:

Study Site:

Projet de Développement de Vaccins Anti-Paludique- Centre, National de Recherche et de Formation sur le Paludisme (PDVAP/CNRFP)
1487 Avenue de l'Oubritenga
01 BP 2208 Ouagadougou 01 Burkina Faso
Sodiomon Bienvenu SIRIMA, BA, MD, PhD
Phone: + 226 50 32 46 95/6
Fax: + 226 50 30 52 20 or + 226 50 31 04 77
s.sirima.cnlp@fasonet.bf

Clinical Laboratory:

Projet de Développement de Vaccins Anti-Paludique- Centre, National de Recherche et de Formation sur le Paludisme (PDVAP/CNRFP)
1487 Avenue de l'Oubritenga
01 BP 2208 Ouagadougou 01 Burkina Faso
Issa NEBIE, MSc, PhD
Laboratory Director
Phone: + 226 50 32 46 95/6
Fax: + 226 50 30 52 20 or + 226 50 31 04 77
issanebie.cnlp@fasonet.bf

Humoral and Cellular (Elispot and Flow Cytometry) Immune Assay Laboratory:

Crucell Holland B.V.
Archimedesweg 4-6
2333 CN Leiden
Stefan Kostense, PhD
Associate Director, Clinical Assays
tel: +31(71)5199224 /fax:+31(71)5199800
Stefan.Kostense@crucell.com

Cellular (whole blood assay) Immune Assay Laboratory:

Projet de Développement de Vaccins Anti-Paludique- Centre, National de Recherche et de Formation sur le Paludisme (PDVAP/CNRFP)
1487 Avenue de l'Oubritenga
01 BP 2208 Ouagadougou 01 Burkina Faso
Issa NEBIE, MSc, PhD
Laboratory Director

Phone: + 226 50 32 46 95/6
Fax: + 226 50 30 52 20 or + 226 50 31 04 77
issanebie.cnlp@fasonet.bf

Data Monitoring:

The EMMES Corporation
401 N. Washington St., Suite 700
Rockville, MD 20850
Tel: 301-251-1161
Fax: 301-251-1355
malaria@emmes.com

Co-Investigator :

LCDR Karl Kronman, MD, MPH, USN

Officer in Charge, Ghana Detachment

Naval Medical Research Unit No. 3

American Embassy, Accra
Mobile: +233-24-4333027
Office: +233-21-741649
karl.kronmann@med.navy.mil

2 BACKGROUND INFORMATION AND SCIENTIFIC RATIONALE

2.1 Background Information

Malaria currently represents one of the most prevalent infections in tropical and subtropical areas throughout the world. Each year, malaria affects around 300 million people and kills 1 to 3 million people in developing countries. Malaria is the leading cause of death in children in sub-Saharan Africa, and drug-resistant parasites and insecticide-resistant vectors increasingly pose challenges for malaria control programs. Other factors include environmental and climatic changes, civil disturbances and increased mobility of populations.

Malaria is caused by the mosquito-borne hematoprotozoan parasites belonging to the genus Plasmodium. Four species of *Plasmodium* protozoa (*P. falciparum, P. vivax, P. ovale* and *P. malariae*) are responsible for the disease in man; many other species cause disease in animals, such as *Plasmodium yoelii* and *Plasmodium berghei* in mice. *P. falciparum* accounts for the majority of infections in man and is the most lethal ("tropical malaria").

Malaria parasites have a life cycle consisting of several stages. Each one of these stages is able to induce specific immune responses against the corresponding stage-specific antigens. Malaria parasites are transmitted to man by several species of female *Anopheles* mosquitoes. Infected mosquitoes inject the sporozoite form of the malaria parasite into the mammalian bloodstream. Before invading hepatocytes, sporozoites remain for a few minutes in the circulation. At this stage the parasite is located in the extracellular environment and is exposed to antibody attack, mainly directed to the circumsporozoite (CS) protein, a major component of the sporozoite surface. Once in the liver, the parasites replicate and develop into schizonts (up to 20,000 per infected cell). During this intracellular stage of the parasite, the main effectors of the host immune response are T lymphocytes, especially CD8+ T cells [1].

Radiation-attenuated sporozoites have been shown to provide protective immunity against malaria, in experimental animal models as well as in humans [2,3]. This vaccine elicits both sporozoite-neutralizing antibodies as well as potent cell-mediated immunity, but it is technically difficult to manufacture [4].

Compelling evidence has been obtained over the last 17 years that CD8+ T cell immunity is as important, if not more important, than B-cell immunity for protection against the liver stages of malaria [5,6,7].

Ad35.CS.01 is a malaria vaccine for which a codon optimized nucleotide sequence representing the *P. falciparum* circumsporozoite (CS) surface antigen is inserted in a replication deficient Adenovirus 35 backbone. It was manufactured by Crucell Holland BV, Leiden, the Netherlands..

2.1.1 Pre-Clinical and Toxicology Studies

New Zealand White Rabbits:

A 99-day toxicology study, adherent to Good Laboratory Practice, was conducted in 60 New Zealand White rabbits using Ad35.CS.01 (manufactured adhering to Good Manufacturing Practice) or placebo given intramuscularly in four IM administrations. Twenty rabbits in each of three cohorts received 1.2 x 10^{10} vp per 1 mL dose of Ad35.CS.01, 1.2 x 10^{11} vp per 1 mL dose of Ad35.CS.01, or 1 mL of placebo consisting of formulation buffer. There was no effect on mortality, clinical observations, cageside observations, dermal Draize observations, body weight changes, ophthalmologic examinations, hematological parameters, coagulation parameters, gross pathology findings, or organ weights. Changes in clinical chemistry parameters were not of clinical significance or not test article related. There was no evidence of systemic toxicity with the exception of a transient effect on food consumption in the high-dose group. Pathology findings included test article-related histological effects seen at the injection sites considered to represent host inflammatory responses to antigen administration.

A 90-day biodistribution study, adherent to Good Laboratory Practice, was conducted with a single 1 mL IM administration of 1.2 x 10^{11} vp of Ad35.CS.01, or placebo, to 30 and 12 New Zealand White rabbits, respectively. The TaqMan qPCR assay used to measure the biodistribution and persistence of the Ad35.CS.01 vaccine demonstrated that Ad35.CS.01 DNA remained primarily localized in the muscle and skin at the injection site with the exception of a few copies detected in the spleens of 6 of 10 rabbits at study day 9. Copy numbers detected in the spleen were 63 and 55 per microgram of genomic DNA, and 4 others that were too low to quantify. The vector did persist at the muscle and skin at the site of injection from study days 9 through 91, but the vector copy number decreased markedly over that period.

Non-human Primates:
A non-GLP (Good Laboratory Practice) immunogenicity and safety study of Ad35.CS was performed in 18 adult rhesus macaques. Seven primates each received 1 x 10^{11} vp of Ad35.CS IM on either a 0, 3 month or 0, 6 month schedule. Four control primates received Ad35 without the transgene as control. Safety assessments included twice daily visual inspections, and clinical examinations of spleen, lymph nodes, joints, and injection site (using the contralateral site for comparison) and measurement of body weight and vital signs on the day of vaccination (day 0) and on days 1, 2, 3, 7, and 14 after each injection.

Clinical laboratory determinations (selected serum chemistries and complete blood counts) were done on the same schedule as the clinical examinations and at specified additional times, which extended for all animals to six months after the last test article injection. These inspections and examinations did not reveal systemic toxicities. Increases in White Blood Cell counts were recorded 24 hours after the first vaccinations in the two test article groups. Occasional elevations were observed in some animals in ALT and GGT over the course of the study, but these elevations were mild, transient, and did not appear to be related to the test article or dosing schedule. At 2 weeks, 3 months, and 6 months after final vaccinations, sera were collected for analysis of CS-specific antibody responses by ELISA and peripheral blood mononuclear cells were harvested for ELISPOT analysis. At all time points and with both assays, Ad35.CS generated significantly higher immune responses than the control agent. Refer to the clinical Investigator's Brochure for details of the immunogenicity responses in the primate study, as well as another study demonstrating immunogenicity of a single IM dose of 1 x 10^9 vp or 1 x 10^{10} vp of Ad35.CS.01 in mice.

2.1.2 Clinical Experience

A Phase 1 randomized, controlled, dosage-escalation trial to evaluate the immunogenicity, safety and reactogenicity in healthy adults 18 to 45 years of age is ongoing in the U.S. Seventy two subjects are randomized in a 5:1 ratio to receive vaccine or normal saline placebo control by the IM route at 0, 1 and 6 months. Fifteen subjects have received vaccine at 10^8 vp/mL, 15 subjects have received vaccine at 10^9 vp/mL, and 15 subjects have received vaccine at 10^{10} vp/mL. Enrollment of subjects receiving vaccine at 10^{11} vp/mL is ongoing.

One SAE, not associated with vaccination, has been reported; it was a small bowel obstruction related to appendectomy for appendicitis. It resolved without sequelae.

All mild and moderate AEs associated with injection resolved without sequelae. A causal association with vaccine cannot be determined, and the safety data have not been unblinded at this time. There were a total of 5 moderate AEs in 4 subjects associated with time of injection: a right sided paresthesia lasting 24 hours, itchy tongue which resolved, brachial plexopathy lasting 24 hours, anemia (iron deficiency) and diaphoresis which resolved the same day.

In the current ongoing study in the U.S., after evaluation of the first 6 participants of Group 10^{11} vp/mL, it was noted that significant reactions (grade 3) occurred in 2 of the 6 and were considered associated with injection. Symptoms began in the first 8 hours following vaccination, but none occurred in the first 4 hours following vaccination. These symptoms included fever, chills, myalgia, nausea and GI side effects such as diarrhea, that occasionally lasted for several days, was self-limited, and may or may not have been related to the vaccine.

Two individuals, one from Group 10^8 vp/mL and one from Group 10^{11} vp/mL, developed Grade 2 signs and symptoms consistent with a brachial plexus neuropathy that was deemed associated with vaccination. For the subject in Group 10^8 vp/mL, symptoms began on the day of vaccination for both the 2nd and 3rd vaccinations but resolved within 24 hours without sequelae. For the subject in Group 10^{11} vp/mL, symptoms began within the first week after 2nd vaccination and were resolved by the Day 7 physical examination. Both of these events resolved without sequelae.

2.2 Rationale

It is hypothesized that the Ad35.CS.01 vaccine will prevent the *P. falciparum* parasite, which causes malaria, from entering and developing within the liver of those who become infected. Ad35.CS.01 would therefore be expected to reduce malaria-attributable morbidity and mortality.

2.3 Potential Risks and Benefits

2.3.1 Potential Risks

Adenovirus Type 35 circumsporozoite vaccine has been administered only in a malaria naïve population in a Phase 1 trial in the US, but not yet in semi-immune human subjects. Therefore, the safety of the vaccine in a population that is semi-immune to malaria has yet to be established. An adenovirus Type 35 vaccine with an insert from *Mycobacterium tuberculosis* has been tested in phase I clinical trials and showed no safety concerns in 94 volunteers immunized to date.

A test-of-concept study of an adenovirus type 5 HIV vaccine (the STEP trial: www.hvtn.org.) has been discontinued for enrollment after the interim analysis showed that vaccine did not prevent HIV infection or change a course of disease. In this trial a potential safety signal has been identified: there were more HIV infections in male participants who received the vaccine than in those who received placebo. Male participants who had higher level of pre-existing antibodies to adenovirus type 5 and engaged in high risk sexual behavior showed a higher incidence of HIV infections. The study was not powered to determine the etiology of these findings. Further analyses are under way to address this potential safety signal.

The risk for participants in this trial (adenovirus type 35 based vector with a malaria gene insert) is theoretically low since it is not clear whether the safety signal is a statistical or biological phenomenon and whether, if shown to be a biological phenomenon, it will apply to other adenoviral vector vaccines, particularly those with non-HIV related inserts, administered to individuals who are not at increased risk of HIV exposure. In addition, Ad35 serotype infection is rare in Africa, and thus, pre-existing immunity is rare.

Additional discomforts of this study are those of having blood drawn from an arm vein, IM injection of the vaccine, and possible reactions to the vaccine. Drawing blood causes transient discomfort and may cause fainting. Infection at the site where blood will be drawn or where the vaccination is given is extremely unlikely, but is a potential risk. Bruising at the site of blood drawing may occur, but can be prevented or lessened by applying pressure for several minutes immediately after the blood draw. Intramuscular injection may cause injection site pain, swelling, and redness. Immediate allergic reactions to vaccine, including anaphylaxis, are in general extremely rare (approximately 1 person in 4,000,000), and might occur as a skin rash such as hives, difficulty breathing, fainting, drop in the blood pressure and death. Such reactions can usually be stopped by emergency medications administered by study personnel. Vaccine recipients may develop systemic reactions such as fever, headaches, body aches, and fatigue. These reactions are usually greatest within the first 24 to 72 hours after vaccination and last 1 to 2 days. Analgesics (*e.g.*, aspirin or Tylenol®) and rest will generally relieve or moderate these symptoms. Other hypersensitivity reactions, including Arthus reactions resulting in large local swelling reactions, are also possible.

In the ongoing Ad35.CS.01 Phase 1 study in the U.S., after evaluation of the first 6 participants of Group D, it was noted that significant reactions (grade 3) occurred in 2 of the 6 and were considered associated with vaccination. Symptoms began in the first 8 hours following vaccination, but none occurred in the first 4 hours following vaccination. These symptoms included fever, chills, myalgia, nausea and GI side effects such as diarrhea, which occasionally lasted for several days, were self-limited, and may or may not have been related to the vaccine. In addition, two individuals, one from Group A and one from Group D, developed signs and symptoms consistent with a Grade 2 brachial plexus neuropathy that was deemed associated with vaccination. For the subject in Group A, symptoms began on the day of vaccination for both the 2nd and 3rd vaccinations but resolved within 24 hours without sequelae. For the subject in Group D, symptoms began within the first week after 2nd vaccination and were resolved by the Day 7 physical examination.

2.3.2 Known Potential Benefits

There are no direct benefits to subjects from participating in the study. However, subjects may benefit altruistically by the knowledge that they are aiding in the malaria vaccine development effort. They may also receive medical attention beyond that which they normally receive, including the screening medical examination, periodic clinical laboratory examinations, and referral for treatment for medical problems that arise during the study. In addition, medical treatment will be provided for malaria illness for all volunteers during the study period, regardless of study-relatedness.

3 OBJECTIVES

3.1 Study Objectives

The primary objective is to assess the safety and reactogenicity of four ascending dosages of Adenovirus 35 circumsporozoite malaria vaccine among healthy, semi-immune, subjects given in 3 doses at 0, 1 and 3 months by IM injection.

The secondary objective is to evaluate the immunogenicity of 3 doses of Adenovirus 35 circumsporozoite malaria vaccine at 4 dosage levels by measuring antibodies against the circumsporozoite antigen by ELISA, neutralizing antibodies against Adenovirus type 35, and T-cell response against the circumsporozoite antigen by cellular immune assays.

3.2 Study Outcome Measures

3.2.1 Primary Outcome Measure

- The number of subjects experiencing severe (Grade 3) solicited injection site reactions within 14 days following vaccination.
- The number of subjects experiencing severe (Grade 3) solicited systemic reactions within 14 days following vaccination.
- The number of subjects spontaneously reporting severe (Grade 3) adverse events considered associated with the vaccination at any point during the study period.
- Serious adverse events considered associated with the vaccination reported at any point during the study period.

3.2.2 Secondary Outcome Measures

- Antibody titers against the malaria circumsporozoite antigen at days 0, 28, 56, 84, 112 and 140 (Geometric Mean titer and individual log ELISA units).
- Neutralizing antibody titers against Adenovirus type 35 by Adenovirus Neutralization Assay at days 0, 28, 56, 84, 112 and 140 (GMT).

3.2.3 Exploratory Outcome Measures

- T cell responses against the malaria circumsporozoite antigen by Elispot at days 0, 28, 56, 84, 112 and 140.
- T cell responses against the malaria circumsporozoite antigen by Flow Cytometry at days 0, 28, 56, 84, 112 and 140.
- T cell responses against the malaria circumsporozoite antigen by a whole blood assays to measure secreted cytokines at days 0, 28, 56, 84, 112 and 140.

4 STUDY DESIGN

The study is a single-center, double-blinded, dosage-escalation clinical trial to assess the safety of four ascending dosages of Adenovirus Type 35 circumsporozoite malaria vaccine administered in 3 doses intramuscularly. We propose to confirm the safety of each dosage of the new vaccine by assessing safety laboratory parameters and reactogenicity for 14 days after the initial dose in the lower dosages before escalating to the next dosage level. Each dosage level group will include 10 subjects given vaccine intramuscularly. Two subjects in each dosage group will receive normal saline as placebo control.

**Group**	**Vaccine Formulation**	**Number of Vaccine/Placebo**
A	Ad35.CS.01 – 10^9 vp/mL	10/2
B	Ad35.CS.01 – 10^{10} vp/mL	10/2
C	Ad35.CS.01 – 5 x 10^{10} vp/mL	10/2
D	Ad35.CS.01 - 10^{11} vp/mL	10/2
Total		40/8

5 STUDY POPULATION

The study will be carried out by the PDVAP/CNRFP in the Saponé health district in the Bazèga province. This rural area is situated 50 km south west of Ouagadougou, the capital city of Burkina Faso in the Sudan-Sahelian eco-climatic zone. The PDVAP/CNRFP has been conducting epidemiological studies in the area last year. A vaccinology unit specifically dedicated for vaccine trials has been built. The PDVAP/CNRFP has further established demographic surveillance system within Saponé health district (87,678 inhabitants living 79 villages around Balonghin). Through this system, data on vital events and basic malariometric parameters have been collected for a deeper and reliable baseline data necessary for potentials malaria phase 1, 2 or 3 vaccines trials.

In this area malaria transmission is markedly seasonal and intense during the rainy season from June to October. The entomological inoculation rate is above one infective bite/person/night during the intense transmission season and drops to 0 during the low season (dry season). The main malaria vectors are *Anopheles gambiae, An. Arabiensis and An. funestus*. *P falciparum* accounts for about 90% of malaria infections. The first line treatment for uncomplicated malaria in Burkina is Artemether-Lumefantrine or Artesunate-Amodiaquine. Bed nets are rarely used in the DSS area with less than 5% of the population owning a net.

5.1 Selection of the study population

From the up to date database of the population in the study area, a list of all adults 18-45 years old from the village of Balonghin and neighbour villages will be drawn. Subjects will be visited at home by field workers who will explain the study to them. They will then be invited to the vaccinology unit based at Balonghin village to receive more information about the study. An educational process will be instituted which will represent several levels of authority starting from the civil authorities to the local village leadership and down to small groups and ultimately individual. Those who provide a signed informed consent will be screened for study eligibility. A total of 48 eligible adults will be enrolled.

In order to establish a bank of PBMC from individuals with known responsiveness against malaria, for use as positive standards in future human cellular assays in studies with malaria vaccine candidates, the informed consent will include a statement that subjects may be approached for an additional blood sampling outside this vaccine trial.

5.2 Enrollment Within Each Dosage Cohort

This study will be performed as a randomized, double-blinded, dosage-escalation study. The first dosage cohort consists of 12 subjects: 10 will receive the Ad35.CS.01 malaria vaccine while 2 will receive placebo control product. The subjects in the first cohort will be enrolled as follows: subject #1 followed by subject #2 with a minimum of a 30 minute waiting period between vaccinating subjects. This schedule will allow for assessment of immediate reactions of subject #1 prior to vaccinating subject #2. Following enrollment of subject #2, a minimum of a 2-day waiting period will be required prior to enrollment of the next four subjects. This schedule will allow for further reactogenicity assessment. The minimum 2-day waiting period will continue between each group of four subjects until the enrollment of 12 subjects into the first cohort has been met.

Following the SMC review of 14-day safety data of the first cohort, the second dosage cohort will contain 12 subjects (10 will receive Ad35.CS.01 and 2 will receive normal saline placebo control product). Six subjects will be enrolled, followed by a minimum of a 2-day waiting period before the next six subjects will be enrolled. The minimum 2 day waiting period will continue between each group of six subjects until the enrollment of 12 subjects into the second cohort has been met. This process will be repeated for the third and fourth cohorts.

Enrollment of additional subjects within any dosage cohort will not occur if a halting rule is met. Enrolled subjects who drop out of the study for any reason will not be replaced.

5.3 Subject Inclusion Criteria

1. Provision of informed consent before any protocol procedures are performed.
2. Males and non-pregnant, non-lactating females between the ages of 18 and 45 years, inclusive.
3. Females and males must agree to practice adequate contraception until at least 28 days following their last immunization dose (including abstinence; hormonal contraception; condoms with spermicidal agents; post-menopausal; or surgical sterilization/vasectomy).
4. Participants must agree to avoid high risk sexual behavior for exposure to HIV.
5. In good health as determined by screening medical history, physical examination (PE), and laboratory assessments.
6. Willingness to comply with protocol requirements.
7. Willingness to be contacted for one year for assessment of serious adverse events.
8. Must be a permanent resident of the Saponé health district (villages around Balonghin) in Burkina Faso.

5.4 Subject Exclusion Criteria

1. Current or recent (within the last four weeks) treatment with parenteral, inhaled, or oral corticosteroids (intranasal steroids are acceptable), or other immunosuppressive agents, or chemotherapy.
2. History of splenectomy.
3. Abnormal screening laboratory values (see Appendix B for laboratory parameters to be tested). Any abnormal screening value for any screening test will exclude the subject from the study. Abnormal screening labs will not be repeated with the exception of high glucose levels will be repeated at a fasting state.
4. History of IV drug abuse.
5. History of, or current medical, occupational, social or family problems as a result of alcohol or illicit drug use.
6. History of moderate to severe mental illness, as defined by symptoms interfering with social or occupational function or suicidal thoughts/attempts.
7. History of receiving blood or blood products (such as blood transfusion, platelet transfusion, immunoglobulins, hyperimmune serum) in the previous 6 months.
8. Vaccination with a live vaccine within the past 30 days or with a nonreplicating, inactivated, or subunit vaccine within the last 14 days.
9. Known hypersensitivity to components of the vaccine
10. History of acute or chronic medical conditions including, but not limited to, disorders of the liver, kidney, lung, heart, or nervous system, or other metabolic or autoimmune/inflammatory conditions.
11. History of coagulation defect or bleeding from (bruising at) multiple sites that cannot be linked to trauma or surgery.
12. History of anaphylaxis or severe hypersensitivity reaction.
13. Severe asthma, as defined by an emergency room visit or hospitalization within the last 12 months.
14. Pregnant or breastfeeding women.
15. Acute illness, including temperature >37.8℃ wi thin one week prior to vaccination.
16. Positive serology for human immunodeficiency virus (HIV), hepatitis C virus (HCV), or hepatitis B surface antigen (HBsAg).
17. Concurrent participation in other investigational protocols or receipt of an investigational product within the previous 30 days or planned receipt of an investigational product within 28 days following the last immunization dose.

18. Identification of any condition that, in the opinion of the investigator, would affect the ability of the subject to understand or comply with the study protocol or would jeopardize the safety or rights of a subject participating in the study.
19. History of malignancy, including hematologic and skin cancers (except for a localized basal cell carcinoma), or known immunodeficiency syndrome.
20. History of previous receipt of a malaria vaccine.
21. **Pre-medication** with analgesic or antipyretic agents in the 6 hours prior to vaccination, or **planned** medication with analgesic or antipyretic in the 24 hours following vaccination. This criterion should not preclude subjects receiving such medication if the need arises.
22. Receipt of a recombinant adenovirus vector vaccine in a prior study.

5.5 Treatment Assignment Procedures

5.5.1 Randomization Procedures

Randomization to either vaccine or placebo will be done online using the enrollment module of The EMMES Corporation's AdvantageEDC^{SM} electronic data capture system. The randomization codes will be included in the enrollment module for the trial. Each subject enrolled into the trial will be assigned a treatment code after demographic and eligibility data have been entered into the system. The site will be provided with a treatment code list to be kept in a secure place with access permitted only to the unblinded pharmacist.

5.5.2 Blinding Procedures

The pharmacist will have exclusive access to the treatment code list. After randomization of the subjects in AdvantageEDC, the pharmacist and assistants will prepare the injection (vaccine or placebo control, according to the treatment code list) in the pharmacy. No subjects or study personnel will be present at the time of preparation. When the injection is ready for administration, the pharmacist will deliver the syringe (covered with tape to mask vaccine), containing the vaccine or placebo control to the vaccinator and the injection will be given to the subject. The pharmacist will be the only unblinded individual involved in the trial. Since the Ad35.CS.01 vaccine and placebo control will not be identical in appearance, the vaccinator will not be involved in the assessment of vaccine reactogenicity. Personnel assessing reactogenicity of the vaccine or laboratory parameters will be blinded. Refer to MOP for a detailed description of vaccine preparation procedures.

5.5.3 Reasons for Withdrawal from Vaccination Schedule

Subjects are free to withdraw from the study at any time. Subjects who have received vaccine or who developed an adverse event or serious adverse event will be encouraged to remain in the study to be followed for safety purposes. A study subject will be discontinued from receiving further investigational product if any clinical adverse event (AE), intercurrent illness, or other medical condition or situation occurs that meets the exclusion criteria (to be reviewed prior to each vaccination), or if continued participation in the study would not be in the best interest of the subject, unless doing so would harm the subject in the opinion of the investigator.

Because a true intent-to-treat analysis requires the inclusion of all participants randomized to the extent possible, this requires an intent-to-treat design in which all participants are followed according to the prespecified schedule with principal, and perhaps secondary, outcome assessments, regardless of compliance, adverse effects, or other post-randomization observations—death and participant refusal excepted.

5.5.4 Handling of Subjects Who Discontinue Vaccination

If, for safety reasons a subject is deemed by the investigators and/or safety monitoring committee (SMC) to be not eligible to receive the study product as per protocol, he/she will discontinue subsequent vaccinations and be followed for safety, reactogenicity and immunogenicity. Subjects who discontinue the vaccinations or terminate their study participation early will not be replaced.

6 STUDY INTERVENTION/INVESTIGATIONAL PRODUCT

6.1 Study Product Description

6.1.1 Acquisition

Vaccine and diluent will be shipped from Crucell to study site. Normal Saline for placebo will be shipped to the investigational pharmacy at the study site from DMID via the DMID Clinical Agent Repository at Fisher BioServices.

6.1.2 Formulation, Packaging, and Labeling

Ad35.CS.01 appears clear to slightly opalescent with no visible particles and is packaged in 3 mL type I glass vials containing 0.75 mL extractable volume with 20 mm rubber injection stoppers. Vials are sealed with a center tear-off aluminum seal. Each vial of drug product (Ad35.CS.01 vaccine) contains Ad **35.CS.01, Tris, Magnesium Chloride, Sodium Chloride, Sucrose, and PolySorbate-80 (non-animal source).**

The diluent appears clear with no visible particles and consists of the formulation buffer alone. The diluent is used to dilute the drug product to the intended dosages for the clinical study.

The diluent contains Tris, Magnesium Chloride, Sodium Chloride, Sucrose, and PolySorbate-80 (non-animal source).

The Normal Saline for use as placebo will be shipped to the investigational pharmacy at the study site from DMID via the DMID Clinical Agent Repository at Fisher BioServices.

6.1.3 Product Storage and Stability

Ad35.CS.01 should be stored at ≤ - 65 °C. Ongoing stability studies at the proposed storage temperature indicate that Ad35.CS.01 is stable for at least 36 months after fill and finish. Storage will be in a monitored and alarmed freezer in the investigational pharmacy. The diluent will be refrigerated at 2 to 8 °C. The normal saline, supplied in single dose vials, will be refrigerated at 2 to 8 °C.

6.2 Dosage, Preparation and Administration of Investigational Product

Instructions for the preparation, dilution, and handling of the assigned dosage form will be provided separately to the investigational pharmacist at the study site. Vaccination will be performed by a trained nurse. Each 0.75 mL dose of the assigned dosage of Ad35.CS.01 or placebo control agent will be administered with a sterile, disposable syringe (masked with tape by the pharmacist) and needle by IM injection into the deltoid muscle. The subject will choose whether the injection will be administered into the right or left deltoid for the first dose. Subsequent injections shall be administered in alternating arms.

6.3 Modification of Investigational Product for a Participant

If, for safety reasons or loss to follow-up, a subject is deemed by the investigators and/or SMC to be ineligible to receive the study product as per protocol, he/she will not receive subsequent vaccinations and will be followed for safety, reactogenicity and immunogenicity. No dose modification will be performed.

6.4 Accountability Procedures for the Study Investigational Products

The study article will be shipped from Crucell to the study site. Use and final disposition of each vial of vaccine or placebo control will be appropriately documented in accordance with ICH GCP (details provided in the MOP). A pharmacist assisted by an assistant pharmacist will be in charge of all these aspects. They will prepare the vaccine, transport it to the field, and manage it locally during the vaccinations sessions. Please see the MOP for additional details.

6.5 Concomitant Medications/Treatments

At each study visit, the subject will be questioned about any concomitant medication use since the previous visit and the information will be recorded. Concomitant medications and treatments that preclude vaccination are listed as exclusion criteria.

7 STUDY SCHEDULE

7.1 Screening Day -42 to -1, Visit 1

- Potential subjects will be provided with a verbal description of the study (purpose and study procedures), and will be asked if they have any questions and to read/sign the consent form. The consent form will be signed prior to the performance of any study procedures.
- The study staff will discuss with the subject his/her medical history, study eligibility criteria and concomitant medication use.
- Vital signs (axillary temperature, blood pressure, pulse) will be obtained.
- A PE will be performed by the investigator (lymph nodes, lungs, heart, liver, spleen) including height and weight. A complete neurologic examination will also be performed, to include assessment of central nerve function, motor function, sensory function, and deep tendon reflexes.
- For females who are capable of bearing children, a urine pregnancy test will be done at screening.
- A 20-mL blood sample will be collected from an arm vein to screen for health as follows:
  - o Hematology: hemoglobin (Hgb), white blood cell count (WBC) with machine differential, absolute neutrophil count (ANC), and platelet count.
  - o Chemistry: glucose, electrolytes (sodium, potassium), alanine aminotranferase (ALT), aspartate aminotransferase (AST), and creatinine.
  - o Serology:, HIV, HCV, and HBsAg.
- A urine sample will be obtained for urinalysis.

7.2 Enrollment/Baseline Day 0, Visit 2

- Eligibility criteria will be reviewed with subjects. Vital signs (axillary temperature, blood pressure, pulse) and interim medical history will be obtained.
- A targeted PE will be performed if indicated by the medical history.
- Concomitant medications will be recorded.
- A urine pregnancy test will be performed, and negative results, within 24 hours prior to vaccination, will be confirmed on all females of childbearing potential.
- A 50-mL blood sample for humoral and cell-mediated immune assays will be collected from a vein in the arm prior to vaccination.

- A thick smear slide will be prepared from the 50ml blood sample and will be stored to be read for parasitemia at a later time if necessary. Refer to section 8.2.1

- Enrolled subjects will be assigned to receive Ad35.CS.01 vaccine or normal saline placebo control using the dosage specified by their group (A→D, sequentially).

- Subjects will be observed in the clinic for a minimum of 30 minutes following vaccination. After 30 minutes, the vaccination site will be examined, vital signs will be obtained, and the subject will be questioned about the presence of any localized or generalized reactogenicity symptoms.
- Any spontaneous AEs that occur will be assessed.

- Subjects will be informed that a field worker will be visiting them at home every day (other than scheduled clinic visit day) for 14 days post each vaccination to record all medical information.

- Subjects will be instructed to return to the study center if they feel febrile on any individual day or if they develop any severe reactions following vaccination.

7.3 Follow-up

7.3.1 Day 1 (+1), 2A, Scheduled home visit

- Study personnel (field workers) will visit the subject at home to solicit local and systemic reactogenicity and concomitant medication information.
- Any spontaneous AEs that have occurred since the last visit will be assessed.

7.3.2 Day 2 (+1), Visit 3

- Subjects will be evaluated in the clinic 2 days after immunization. Vital signs (axiliary temperature, blood pressure, pulse) will be taken, and information regarding systemic and local reactions will be solicited and recorded. An examination of the vaccination site will be performed.
- Any spontaneous AEs that have occurred since the last visit will be assessed..

- A PE will be performed, to include an assessment of lymph nodes, lungs, heart, liver, and spleen. A complete neurologic examination will also be performed, to include assessment of motor function, sensory function, and deep tendon reflexes.

- Obtain subject’s interim medical history.

- Concomitant medications will be reviewed and updated, if applicable.
- Subjects will be reminded about scheduled home visits during which field workers record auxiliary temperatures and local and systemic reactions for the 14-day period after vaccination.
- Subjects will be reminded to come back to the study center if they develop any severe reactions (grade 3) during the study.

7.3.3 Day 7 (± 2), Visit 4

- Subjects will be evaluated in the clinic 7 days after immunization. Vital signs (axiliary temperature, blood pressure, pulse) will be taken, and information regarding systemic and local reactions will be solicited and recorded. An examination of the vaccination site will be performed.
- Any spontaneous AEs that have occurred since the last visit will be assessed.
- A PE will be performed, to include an assessment of lymph nodes, lungs, heart, liver, and spleen. A complete neurologic examination will also be performed, to include assessment of motor function, sensory function, and deep tendon reflexes.
- Obtain subject's interim medical history.
- Concomitant medications will be reviewed and updated, if applicable.
- A 10-mL blood sample will be collected for safety laboratory (hematology, chemistry) testing. A urine sample will also be obtained for urinalysis.
- Subjects will be reminded about scheduled home visits during which field workers record auxiliary temperatures and local and systemic reactions for the 14-day period after vaccination.
- Subjects will be reminded to come back to the study center if they develop any severe reactions during the study.

7.3.4 Day 14 (± 3), Visit 5

- Subjects will be evaluated in the clinic 14 days after immunization. Vital signs (auxiliary temperature, blood pressure, pulse) will be assessed and recorded. Information regarding local and systemic reactions will be solicited and recorded. An examination of the vaccination site will be performed.

- Any spontaneous AEs that have occurred since the last visit will be assessed.
- A targeted PE will be performed if indicated by the subject's interim medical history. A complete neurologic examination will also be performed, to include assessment of motor function, sensory function, tone, and deep tendon reflexes.
- Concomitant medications will be reviewed and updated, if applicable.

7.3.5 Day 28 (window period: -3 days to +3 days), Visit 6, 2nd vaccination

- Eligibility criteria will be reviewed with subjects. Vital signs (axiliary temperature, blood pressure, pulse) and interim medical history will be obtained.
- Any spontaneous AEs that have occurred since the last visit will be assessed.
- A targeted PE will be performed if indicated by the subject's interim medical history. A complete neurologic examination will also be performed, to include assessment of motor function, sensory function, and deep tendon reflexes.
- Concomitant medications will be reviewed and updated, if applicable.
- A urine pregnancy test will be performed, and negative results, within 24 hours prior to vaccination, will be confirmed on all females of childbearing potential
- A 50-mL blood sample for humoral and cell-mediated immune assays will be collected from a vein in the arm prior to vaccination.
- A thick smear slide will be prepared from the 50ml blood sample and will be stored to be read for parasitemia at a later time if necessary. Refer to section 8.2.1.
- Enrolled subjects will receive Ad35.CS.01 vaccine or normal saline placebo control at the same dosage used on Day 0.
- Subjects will be observed in the clinic for a minimum of 30 minutes following vaccination. After 30 minutes, the vaccination site will be examined, vital signs will be obtained, and the subject will be questioned about the presence of any localized or systemic reactions.
- Subjects will be informed that a field worker will be visiting them at home during 14 days post each vaccination to record all medical information.
- Subjects will be instructed to come back to the study center if they feel febrile on any individual day or if they develop any severe reactions following vaccination.

7.3.6 Day 29 (+1), post-2nd vaccination, Visit 6A, Scheduled home visit

- Study personnel (field worker) will visit the subject at home to solicit local and system reactions and concomitant medication information.
- Any spontaneous AEs that have occurred since the last visit will be assessed.

7.3.7 Day 30 (+1), post-2nd vaccination, Visit 7

- Subjects will be evaluated in the clinic 2 days after their second immunization. Vital signs (axiliary temperature, blood pressure, pulse) will be taken, and information regarding systemic and local reactions will be solicited and recorded. An examination of the vaccination site will be performed
- Any spontaneous AEs that have occurred since the last visit will be assessed.
- A PE will be performed, to include an assessment of lymph nodes, lungs, heart, liver, and spleen. A complete neurologic examination will also be performed, to include assessment of motor function, sensory function, and deep tendon reflexes.
- Obtain subject's interim medical history.
- Concomitant medications will be reviewed and updated, if applicable.
- Subjects will be reminded about scheduled home visits during which field workers record their auxiliary temperatures, local and systemic reactions for the 14-day period after vaccination.
- Subjects will be reminded to come back to the study center if they develop any severe reactions during the study.

7.3.8 Day 35 (±2), post-2nd vaccination, Visit 8

- Subjects will be evaluated in the clinic 7 days after their second immunization. Vital signs (auxiliary temperature, blood pressure, pulse) will be taken, and information regarding systemic and local reactions will be solicited and recorded. An examination of the vaccination site will be performed.
- Any spontaneous AEs that have occurred since the last visit will be assessed.
- A PE will be performed, to include an assessment of lymph nodes, lungs, heart, liver, and spleen. A complete neurologic examination will also be performed, to include assessment of motor function, sensory function, and deep tendon reflexes.

- Obtain subject’s interim medical history.
- Concomitant medications will be reviewed and updated, if applicable.
- A 10-mL blood sample will be collected for safety laboratory (hematology, chemistry) testing. A urine sample will also be obtained for urinalysis.
- Subjects will be reminded about scheduled home visits during which field workers record their auxiliary temperatures, local and systemic reactions for the 14-day period after vaccination.
- Subjects will be reminded to come back to the study center if they feel febrile or if they develop any severe (grade 3) reactions during the study.

7.3.9 Day 42 (±3), post-2nd vaccination, Visit 9

- Subjects will be evaluated in the clinic 14 days after their second immunization. Vital signs (auxiliary temperature, blood pressure, pulse) will be assessed and recorded. Information regarding local and systemic reactions will be solicited and recorded. An examination of the vaccination site will be performed.
- Any spontaneous AEs that have occurred since the last visit will be assessed.
- A targeted PE will be performed if indicated by the subject’s interim medical history. A complete neurologic examination will also be performed, to include assessment of motor function, sensory function, and deep tendon reflexes.
- Concomitant medications will be reviewed and updated, if applicable.

7.3.10 Day 56 (±3), post-2nd vaccination, Visit 10

- Subjects will be evaluated in the clinic 28 days after their second immunization. Vital signs (auxiliary temperature, blood pressure, pulse) will be assessed and recorded. An examination of the vaccination site will be performed.
- Any spontaneous AEs that have occurred since the last visit will be assessed.
- A targeted PE will be performed if indicated by the subject’s interim medical history. A complete neurologic examination will also be performed, to include assessment of motor function, sensory function, and deep tendon reflexes.
- Concomitant medications will be reviewed and updated, if applicable.

- A 50-mL blood sample will be collected for humoral and cell-mediated immune assays.

7.3.11 Day 84 (±3), post-2nd vaccination, Visit 11, 3rd vaccination

- Eligibility criteria will be reviewed with subjects. Vital signs (axillary temperature, blood pressure, pulse) and interim medical history will be obtained.
- Any spontaneous AEs that have occurred since the last visit will be assessed.
- A targeted PE will be performed if indicated by the subject's interim medical history. A complete neurologic examination will also be performed, to include assessment of motor function, sensory function, and deep tendon reflexes.
- Concomitant medications will be reviewed and updated, if applicable.
- A urine pregnancy test will be performed, and negative results, within 24 hours prior to vaccination, will be confirmed on all females of childbearing potential
- A 50-mL blood sample for humoral and cell-mediated immune assays will be collected from a vein in the arm prior to vaccination.
- A thick smear slide will be prepared from the 50ml blood sample and will be stored to be read for parasitemia at a later time if necessary. Refer to section 8.2.1.
- Enrolled subjects will receive Ad35.CS.01 vaccine or normal saline placebo control at the same dosage used on Day 0.
- Subjects will be observed in the clinic for a minimum of 30 minutes following vaccination. After 30 minutes, the vaccination site will be examined, vital signs will be obtained, and the subject will be questioned about the presence of any localized or generalized reactogenicity symptoms.
- Subjects will be informed that a field worker will be visiting them at home during 14 days post each vaccination to record all medical information.
- Subjects will be instructed to come back to the study center if they feel febrile on any individual day or if they develop any severe (grade 3) reactions following vaccination.

7.3.12 Day 85 (+1), post-3rd vaccination, Visit 11 A, Scheduled home visit

- Study personnel (field workers) will visit the subject at home to solicit local and systemic reactions and concomitant medication information.

- Any spontaneous AEs that have occurred since the last visit will be assessed.

7.3.13 Day 86 (+1), post-3rd vaccination, Visit 12

- Subjects will be evaluated in the clinic 2 days after their third immunization. Vital signs (auxiliary temperature, blood pressure, pulse) will be taken, and information regarding systemic and local reactions will be solicited and recorded. An examination of the vaccination site will be performed.
- Any spontaneous AEs that have occurred since the last visit will be assessed.
- A PE will be performed, to include an assessment of lymph nodes, lungs, heart, liver, and spleen. A complete neurologic examination will also be performed, to include assessment of motor function, sensory function, and deep tendon reflexes.
- Obtain subject's interim medical history.
- Concomitant medications will be reviewed and updated, if applicable.
- Subjects will be reminded about the scheduled home visits during which field workers record their auxiliary temperatures, local and systemic reactions for the 14-day period after vaccination.
- Subjects will be reminded to come back to the study center if they develop any severe reactions (grade 3) during the study.

7.3.14 Day 91 (±2), post-3rd vaccination, Visit 13

- Subjects will be evaluated in the clinic 7 days after their third immunization. Vital signs (auxiliary temperature, blood pressure, pulse) will be taken, and information regarding systemic and local reactions will be solicited and recorded. An examination of the vaccination site will be performed.
- Any spontaneous AEs that have occurred since the last visit will be assessed.
- A PE will be performed, to include an assessment of lymph nodes, lungs, heart, liver, and spleen. A complete neurologic examination will also be performed, to include assessment of motor function, sensory function, and deep tendon reflexes.
- Obtain subject's interim medical history.

- Concomitant medications will be reviewed and updated, if applicable.
- A 10-mL blood sample will be collected for safety laboratory (hematology, chemistry) testing. A urine sample will also be obtained for urinalysis .
- Subjects will be reminded about scheduled home visits during which field workers record their auxiliary temperatures, local and systemic symptoms for the 14-day period after vaccination.
- Subjects will be reminded to come back to the study center if they develop any severe (grade 3) reactions during the study.

7.3.15 Day 98 (±3), post-3rd vaccination, Visit 14

- Subjects will be evaluated in the clinic 14 days after their third immunization. Vital signs (auxiliary temperature, blood pressure, pulse) will be assessed and recorded. Information regarding local and systemic reactions will be solicited and recorded. An examination of the vaccination site will be performed.
- Any spontaneous AEs that have occurred since the last visit will be assessed.
- A targeted PE will be performed if indicated by the subject's interim medical history. A complete neurologic examination will also be performed, to include assessment of motor function, sensory function, and deep tendon reflexes.
- Concomitant medications will be reviewed and updated, if applicable.

7.3.16 Day 112 (±3), post-3rd vaccination, Visit 15

- Subjects will be evaluated in the clinic 28 days after their third immunization. Vital signs (auxiliary temperature, blood pressure, pulse) will be assessed and recorded. An examination of the vaccination site will be performed.
- Any spontaneous AEs that have occurred since the last visit will be assessed.
- A targeted PE will be performed if indicated by the subject's interim medical history. A complete neurologic examination will also be performed, to include assessment of motor function, sensory function, and deep tendon reflexes.
- Concomitant medications will be reviewed and updated, if applicable.
- A 50-mL blood sample will be collected for humoral and cell-mediated immune assays.

7.3.17 Day 140 (±1 month), Visit 16

- Subjects will be evaluated in the clinic. Vital signs (auxiliary temperature, blood pressure, pulse) will be assessed and recorded. Subjects will be interviewed regarding any potential risk behavior that would increase their chance of becoming infected with HIV virus.
- Any spontaneous AEs that have occurred since the last visit will be assessed.
- A targeted PE will be performed if indicated by the subject's interim medical history.
- Concomitant medications will be reviewed and updated, if applicable.
- A 50-mL blood sample will be collected for humoral and cell-mediated immune assays.

7.3.18 Day 196 (±1 month), Visit 17

- Subjects will be evaluated in the clinic. Vital signs (auxiliary temperature, blood pressure, pulse) will be assessed and recorded. Subjects will be interviewed regarding any potential risk behavior that would increase their chance of becoming infected with HIV virus.
- Any spontaneous AEs that have occurred since the last visit will be assessed.
- A targeted PE will be performed if indicated by the subject's interim medical history.
- Concomitant medications will be reviewed and updated, if applicable.

7.3.19 Day 252 (+1 month), Visit 18

- Subjects will be evaluated in the clinic. Vital signs (auxiliary temperature, blood pressure, pulse) will be assessed and recorded. Subjects will be interviewed regarding any potential risk behavior that would increase their chance of becoming infected with HIV virus.
- Any spontaneous AEs that have occurred since the last visit will be assessed.
- A targeted PE will be performed if indicated by the subject's interim medical history.
- Concomitant medications will be reviewed and updated, if applicable.

7.3.20 Day 308 (+1 month), Visit 19

- Subjects will be evaluated in the clinic. Vital signs (auxiliary temperature, blood pressure, pulse) will be assessed and recorded. Subjects will be interviewed regarding any potential risk behavior that would increase their chance of becoming infected with HIV virus.
- Any spontaneous AEs that have occurred since the last visit will be assessed.
- A targeted PE will be performed if indicated by the subject's interim medical history.
- Concomitant medications will be reviewed and updated, if applicable.

7.3.21 Day 365 (+1 month), Visit 20

- Subjects will be evaluated in the clinic. Vital signs (auxiliary temperature, blood pressure, pulse) will be assessed and recorded. Subjects will be interviewed regarding any potential risk behavior that would increase their chance of becoming infected with HIV virus.
- Any spontaneous AEs that have occurred since the last visit will be assessed.
- A targeted PE will be performed if indicated by the subject's interim medical history.
- Concomitant medications will be reviewed and updated, if applicable.
- A 5-mL blood sample will be collected for HIV testing.

7.4 Early Termination

Subjects may be discontinued from follow-up if any of the following criteria are met:

- Death
- Serious illness or disability making it impossible to maintain follow-up
- Lost to follow-up
- Subject choice
- Termination of the study

- Any circumstance in the opinion of the investigator that would prevent the subject from completing follow-up or that would put the subject at risk.

7.5 Early Termination Visit

If a subject is terminated from the study early, when applicable, every effort should be made to perform the following procedures:

- Review current health status and note any changes since the last visit.
- Record all concomitant medications.
- Obtain 10-mL blood sample for safety laboratory testing (hematology, chemistry).
- Obtain 50-mL blood sample for the humoral and cell-mediated immune assays.
- Obtain 5-mL blood sample for HIV testing.
- Obtain urine specimen for urinalysis.
- Perform a targeted physical examination, as indicated.
- Solicit information regarding AEs. Any ongoing related AEs will be followed to resolution or until a stable chronic condition has been established.
- Subjects will be encouraged to permit continued follow-up of AEs if possible.

7.6 Unscheduled Visit

Subjects may be asked to come in for additional clinic visits if the need arises for follow-up of local or systemic AE such as neurologic or febrile illnesses. Neurological symptoms will be evaluated with a full neurological exam and neurological consultation if needed. Any febrile illness will be evaluated as necessary to identify the etiology following standard procedures at the site.

A supplemental visit source document will be filled and signed by the appropriate personnel. All unscheduled visits will be entered into the EMMES Corporation AdvantageEDC^{SM} internet data entry system on the appropriate eCRFs. Please see the Manual of Procedures for details.

8 STUDY PROCEDURES/EVALUATIONS

8.1 Clinical Evaluations

Medical history will be obtained by interview of the subjects. Subjects will be queried regarding a history of significant medical disorders of the head, eyes, ears, nose, throat, mouth, cardiovascular system, lungs, gastrointestinal tract, liver, pancreas, kidney, nervous system, blood, lymph glands, endocrine system, musculoskeletal system, skin, and genital/reproductive tract. A history of any allergies, cancer, immunodeficiency, psychiatric illness, substance abuse, and autoimmune disease will be solicited.

All current medications (prescription, over-the-counter drugs, vitamins, and supplements) will be recorded. Assessment of eligibility also will include a review of permitted and prohibited medications (per the inclusion/exclusion criteria).

A physical examination will be conducted to include an assessment of lymph nodes, lungs, liver, heart, and spleen. A full neurological examination will also be conducted. Axillary temperature, pulse and blood pressure will be determined before immunization.

A targeted PE will be conducted on routine visit dates. The extent of this PE will be based on any reported AEs or reactogenicity events or to follow up on previously reported AEs.

Solicited reactogenicity assessments will include a brief history for assessment of AEs and a targeted physical examination, which includes an assessment of erythema, induration, pain/tenderness, and ecchymosis at the injection site.

The subject will be interviewed about solicited local and systemic reactogenicity and spontaneous AEs, and the data transcribed into the case report form (CRF).

8.2 Laboratory Evaluations

8.2.1 Clinical Laboratory Evaluations

- Hematology: hemoglobin (Hgb), white blood cell count (WBC) with machine differential, absolute neutrophil count (ANC), and platelet count (5 mL blood).
- Chemistry: glucose, electrolytes (sodium, potassium), alanine aminotranferase (ALT), aspartate aminotransferase (AST), and creatinine (5 mL blood).
- Serology: HIV, HBsAg, HCV antibodies, (10 mL blood).

- Urinalysis: dipstick for blood and protein and urine β-HCG prior to each vaccination.
- A thick smear slide will be prepared prior to each vaccination and will be stored to be read for parasitemia at a later time in the event that a safety concern arises regarding a possible association between vaccination and concomitant asymptomatic parasitemia.

8.2.2 Humoral and Cell-mediated Immune Assays

To assess both humoral and cell-mediated immune responses to the vaccine, 50 mL of blood will be drawn from subjects on the following study days: 0, 28, 56, 84, 112, 140 days. From the 50 mL blood for immunological responses, 10mL will be used for serum isolation and subsequent humoral assays, and 40 mL blood will be used for cellular assays: 30mls for PBMC isolation and 10 ml for whole blood stimulation followed by cytokine ELISA . Complete information on all immunological assays, including methods, reference standards, and performance sites are included in the Manual of Procedures.[1]

8.2.2.1 Humoral Assays

Ten ml of blood will be collected per draw for humoral assays at 6 time points: 1) day 0 prior to the 1st vaccination, 2) just prior to the 2nd vaccination, 3) 28 days after the 2nd vaccination, 4) just prior to the 3rd vaccination, and 5) 28 days after the 3rd vaccination, 6) 56 days after 3rd vaccination. The assays to be performed include an ELISA to determine antibody titers against the circumsporozoite antigen and an Adenovirus Neutralization Assay for neutralizing antibody titers against Adenovirus type 35, and possibly other relevant Adenovirus serotypes (*e.g.* Adenovirus type 5).

8.2.2.2 Cellular Assays

Blood draws for assessment of cellular immune response will be performed at six time points: 1) day 0 prior to the 1st vaccination, 2) just prior to the 2nd vaccination, 3) 28 days after the 2nd vaccination, 4) just prior to the 3rd vaccination, and 5) 28 days after the 3rd vaccination, 6) 56 days after 3rd vaccination. Per blood draw, 40 mL of blood will be used for cellular assays, of which 30 mL is used for PBMC isolation and 10 ml for whole blood stimulation followed by cytokine ELISA. Blood volumes were determined to ensure sufficient numbers of cells to establish stimulation protocols and evaluate cell-mediated immune responses. Cellular assays will be performed when the first 6 samples have been collected with samples randomized over assay runs to distribute variation over all groups and timepoints.

[1] For description of the Adenovirus Neutralization Assay, see Sprangers, MC et al. Quantifying Adenovirus Neutralizing Antibodies by Luciferase Transgene Detection: addressing preexisiting immunity to vaccine and gene therapy vectors. J. Clin. Microbiol. 41(11):5046, 2003.

Although T cell responses are optimally examined using freshly isolated cells, examining fresh samples collected at different time points is not possible. It has been shown that both $CD4^+$ and $CD8^+$ T cells are readily detected in frozen samples. Thus, Elispot and Intracellular Cytokine Staining (ICS) will be performed on cryopreserved peripheral blood mononuclear cells. Characterization of antigen-specific T cell responses will be evaluated based on the quantity and the phenotype of responding cells to a specific antigenic stimulus using an Elispot assay as the primary quantitative readout and an IFN-γ ICS assay to preliminarily assess the CD4+ vs. CD8+ T cell response. The ICS assay will include the phenotypic markers CD4, CD8, and CD3 and intracellular detection of IFN-γ. Additional markers may be included as feasible. In addition whole blood assay will also be used to measure secreted cytokines production (IFN-γ and IL10) in culture supernatant.

Stimulation of cells in the Elispot, ICS, and whole blood assays will utilize an overlapping peptide matrix. A pool of 15-mer peptides was acquired which overlap by 11 amino acids spanning the entire length and corresponding to the amino acid sequence of the circumsporozoite protein used in the vaccine.

8.2.3 Specimen Preparation, Handling, and Shipping

Instructions for Specimen Preparation, Handling, and Storage

Samples for Cellular Assays – All samples will be drawn into sodium heparin anticoagulant green top Vacutainer tubes. Tubes will be rocked several times immediately after drawing to ensure that blood does not form microclots. Each tube will be labeled with the study identifier, subject number, visit number and date, and processed within 2 hours. Of the 50 mL blood drawn, 30 mL will be used for PBMC isolation and cryopreservation and 10ml will be used for whole blood assays. Frozen samples will be shipped to Crucell/repository for analysis in IFN-γ Elispot and Flow Cytometry. PBMC will be isolated from whole blood, cryopreserved at ≤-65°C in a freezer,, and stored in a liquid nitrogen freezer in the vapor phase.

Samples for humoral immune assays – Of the 50 mL blood drawn, 10 mL will be used for humoral immune assays. Samples will be drawn into serum separator tubes. Following collection, blood will be allowed to sit between 30 and 60 minutes. Then it will be centrifuged in the Vacutainer tube according to the manufacturer’s specifications. Under sterile conditions, the serum will be collected and aliquoted according to study procedures. Labeled aliquots will be placed in the appropriate fiberboard cryoboxes. The filled cryoboxes will be stored at ≤-65°C in a freezer that is monitored.

Specimen Shipment

For analysis of the humoral response, two serum aliquots of each blood draw will be shipped to Crucell BV, Leiden, Netherlands and pending shipment failure, the remainder of the specimens will be stored at CNRFP. If additional storage is necessary, specimens may be shipped to Fishers Repository. For analysis of the cellular immune response, PBMC specimens will be shipped to Crucell BV, Leiden, Netherlands in two separate shipments. Whole blood assays will be processed at CNRFP/PDVAP laboratory in Ouagadougou, Burkina Faso.

9 ASSESSMENT OF SAFETY

9.1 Methods and Timing for Assessing, Recording, and Analyzing Safety Parameters

Safety will be assessed by frequency and incidence of AEs and SAEs in each dosage group. A safety monitoring committee (SMC) will be convened by DMID to review safety information from study subjects. See also section 9.4.

9.1.1 Adverse Events, Reactogenicity, Serious Adverse Events

The investigator is responsible for reporting all AEs that are observed or reported during the study, regardless of their relationship to study product. Initial vaccine reactions will be assessed for at least 30 minutes after vaccination. Subjects will be visited by the study personnel to record both solicited vaccine reactions and any unsolicited AEs. Solicited local and systemic reactions and spontaneous AEs will be collected for 14 days following vaccination.

Solicited systemic reactions will include the following: headache, malaise, myalgia, fever, chills, nausea and vomiting. Solicited local injection site reactions will include the following: induration or swelling, erythema (redness), pain/tenderness, and ecchymosis or bruising.

Definition of Adverse Event

Adverse Event: International Conference on Harmonisation (ICH) guideline E6(R1) defines an AE as any untoward medical occurrence in a clinical investigation subject administered a pharmaceutical product regardless of its causal relationship to the study treatment. An AE can therefore be any unfavorable and unintended sign (including any clinically significant abnormal laboratory finding), symptom, or disease temporally associated with the use of medicinal (investigational) product. The occurrence of an AE may come to the attention of study personnel during study visits and interviews or by a vaccine recipient presenting for medical care.

All AEs must be graded for severity and relationship to study product. Adverse events characterized as intermittent require documentation of onset and duration of each episode.

Severity of Event: All AEs will be assessed by a licensed clinician (*i.e.* medical doctor, nurse practitioner, or physician's assistant) using a protocol-defined grading system. For events not included in the protocol-defined grading system, the following guidelines will be used to quantify severity.

- Mild: events require minimal or no treatment and do not interfere with the subject's daily activities;
- Moderate: events result in a low level of inconvenience or concern with the therapeutic measures. Moderate events may cause some interference with functioning;
- Severe: events interrupt a subject's usual daily activity and may require systemic drug therapy or other treatment. Severe events are usually incapacitating; or
- Life-threatening: Any adverse drug experience that places the subject in the view of the Investigator, at immediate risk of death from the reaction as it occurred, ie, it does not include a reaction that had it occurred in a more severe form, might have caused death.

Relationship to study products/vaccines: The investigator's assessment of the relationship of an AE to study drug/vaccine is part of the documentation process, but it is not a factor in determining what is or is not reported in the study. If there is any doubt as to whether a clinical observation is an AE, the event should be reported. All AEs must have their possible relationship to study vaccine assessed using the following terms: associated or not associated. In a clinical trial, the study product must always be suspect. To help assess relationship, the following guidelines are used.

- **Associated –** There is a suspicion that there is an association between the study product and the AE, the event is temporally related to the administration of the study product, and no other etiology explains the event.
- **Not Associated –** There is no suspicion that there is an association between the study product and the AE, the event is temporally independent of the study product; and/or the event appears to be explained by another etiology.

Local Reactions – Injection Site Reactogenicity

Reactogenicity events are AEs that are known to occur with this type of vaccine. Reactogenicity will be analyzed using the following grading systems:

Local Reaction	Mild (Grade 1)	Moderate (Grade 2)	Severe (Grade 3)
Pain/Tenderness	Does not interfere with activity	Interferes with activity	Prevents daily activity
Erythema/Redness*	>0 – 5 cm and does not interfere with activity	5.1 – 10 cm or interferes with activity	>10 cm or prevents daily activity
Induration/Swelling*	>0 – 5 cm and does not interfere with activity	5.1 – 10 cm or interferes with activity	>10 cm or prevents daily activity
Ecchymosis/Bruising*	>0 – 5 cm and does not interfere with	5.1 – 10 cm or interferes with activity	>10 cm or prevents daily activity

	activity		

* Will be measured at the greatest single diameter

Systemic Reactogenicity Events

Systemic events include signs, symptoms, and laboratory tests. These may be solicited or spontaneously reported. Grading of systemic events are as follows:

Systemic Signs: An axillary temperature of 37.5C is considered fever in adults. Fever severity will be graded as follows:

	**Mild (Grade 1)**	**Moderate (Grade 2)**	**Severe (Grade 3)**
Fever	37.8C (100˚F) - 38.4℃ (101.1 ˚F)	38.5℃ (101.2 ˚F) - 38.9℃ (102.1 ˚F)	>38.9℃ (102.1 ˚F)

Systemic symptoms

The following grading system will be used in evaluating the subjective systemic events:

**Reaction**	**Mild (Grade 1)**	**Moderate (Grade 2)**	**Severe (Grade 3)**
Headache	Does not interfere with activity	Interferes with activity	Prevents daily activity
Malaise	Does not interfere with activity	Interferes with activity	Prevents daily activity
Myalgia	Does not interfere with activity	Interferes with activity	Prevents daily activity
Chills	Does not interfere with activity	Interferes with activity	Prevents daily activity
Nausea	Does not interfere with activity	Interferes with activity	Prevents daily activity
Vomiting	Does not interfere with activity	Interferes with activity	Prevents daily activity

Laboratory testing: Laboratory testing will be performed on the specified study days and graded according to the scale listed in Appendix B.

Serious Adverse Event

An SAE is defined as an AE meeting one of the following conditions:

- Results in death during the period of protocol defined surveillance.
- Is life threatening (defined as a subject at immediate risk of death at the time of the event).

- Requires inpatient hospitalization or prolongation of existing hospitalization during the period of protocol defined surveillance.
- Results in congenital anomaly or birth defect.
- Results in a persistent or significant disability/incapacity.
- Any other important medical event that may not result in death, be life threatening, or require hospitalization, may be considered an SAE when, based upon appropriate medical judgment, the event may jeopardize the subject and may require medical or surgical intervention to prevent one of the outcomes listed above.

- Examples of such medical events include allergic bronchospasm requiring intensive treatment in an emergency room or at home, blood dyscrasias or convulsions that do not result in inpatient hospitalization, the development of drug dependency or drug abuse or a diagnosis of HIV infection.

9.2 Reporting Procedures

Solicited local and systemic reactions and spontaneously reported adverse events will be captured on the appropriate CRF.

Information to be collected for spontaneous AEs includes event description, date of onset, investigator assessment of severity, investigator assessment of relationship to study product, date of resolution of the event, seriousness, and outcome. The severity of non-serious AEs may be assessed by a licensed clinician (i.e., medical doctor, nurse practitioner, physician's assistant) listed on the Form FDA 1572. The causal relationship of non-serious AEs can be assessed by a licensed clinician (*i.e.* medical doctor, nurse practitioner, physician's assistant). All AEs occurring while on study will be documented appropriately regardless of relationship to the Study Vaccine. All AEs will be followed to adequate resolution.

Any medical condition that is present at screening will be considered as baseline and will not be reported as an AE. If the severity of any preexisting medical condition increases during the study period, then it will be recorded as an AE.

9.2.1 Serious Adverse Event Detection and Reporting

All SAEs will be:

- Assessed for severity and causal relationship by a physician listed on the Form FDA 1572 as the principal investigator (PI) or subinvestigator.
- Recorded on the appropriate SAE report form.

- Assessed by an Independent Safety Monitor (ISM) that will share his/her assessment with DMID.
- Followed through resolution by a study physician.
- Reviewed by the safety monitor, the SMC (periodic review unless associated), DMID, and the IRB.

Any AE considered serious by the PI or subinvestigator or that meets the aforementioned criteria must be submitted on an SAE form to DMID Pharmacovigilance Group, at the following address:

DMID Pharmacovigilance Group
Clinical Research Operations and Management Support (CROMS)
6500 Rock Spring Dr. Suite 650
Bethesda, MD 20814, USA
SAE Hot Line: 1-800-537-9979 (US) or 1-301-897-1709 (outside US)
SAE FAX Phone Number: 1-800-275-7619 (US) or 1-301-897-1710 (outside US)
SAE Email Address: PVG@dmidcroms.com

(Note: These are not toll-free numbers, site will have to apply any directions required by their local phone company).

Questions about SAE reporting may be referred to the SAE Hotline at 800-201-8725.

In addition to the SAE form, selected SAE data fields must also be entered into the EMMES Corporation AdvantageEDC^{SM} web-based data entry system. Refer to the Manual of Procedures for details regarding this procedure. Timelines for submission of an SAE form are as follows:

- All deaths and life-threatening events, regardless of relationship, will be recorded on the SAE form and sent by fax within 24 hours of site awareness of the death or event.
- All other SAEs, regardless of whether they are associated or not, need to be reported to DMID within 24 hours of you becoming aware of them.

Other supporting documentation of the event may be requested by the pharmacovigilance contractor and should be provided as soon as possible.

All SAEs will be followed until satisfactory resolution or until the PI or subinvestigator deems the event to be chronic or the subject to be stable.

9.2.2 Regulatory Reporting for Studies Conducted Under DMID-Sponsored Investigational New Drug (IND) Application

Following notification from the investigator, DMID, the investigational new drug (IND) sponsor, will report events that are both serious and unexpected events that are related to study

product(s) to the FDA within the required timelines as specified in 21 CFR Part 312 Section 312.32: fatal and life-threatening events within 7 calendar days (by telephone or fax). All written reports will be sent within 15 calendar days. All SAEs designated as "not related" to study product(s) will be reported to the FDA at least annually in a summary format.

9.2.3 Reporting of Pregnancy

Pregnancies occurring in study subjects, i.e., any pregnancy that occurs within 3 months after the last vaccination, will be documented on the Pregnancy Reporting From provided by EMMES, and this form will be used for the pregnancy event data entry into AdvantageEDC^{SM}. All study-mandated blood samples will be obtained and the subject will continue in follow-up for safety events. Pregnancies will be followed to pregnancy outcome pending the subject's or their partner's permission.

9.2.4 Type and Duration of the Follow-up of Subjects after Adverse Events

Adverse events will be followed until resolved or considered stable.

9.3 Halting Rules

Subject safety data will be reviewed on an ongoing basis by EMMES Corporation and through the AE reporting system. Reactogenicity and safety laboratory data will be entered into the EMMES Corporation AdvantageEDC^{SM} at the study site within 2 business days of data acquisition (*e.g.*, receiving the laboratory reports). If any of the following halting rules criteria are met after vaccination, then enrollment and vaccinations will be stopped and data will be reviewed. The EMMES Corporation data management personnel will monitor the occurrence of the events listed below and notify the investigators and NIH/NIAID/DMID if any of the stopping criteria are met. The DMID Medical Monitor, in consultation with the investigators and site ISM, will determine whether the SMC should review the event. When directed by DMID, The EMMES Corporation and study investigators will provide information relevant to the event and affected individual to the SMC electronically for a rapid review/assessment to be completed within 24 hours. The SMC will provide feedback regarding the relatedness to vaccine and recommendations regarding how to proceed (e.g., continue enrollment and vaccinations without further review, formal halt and/or *ad hoc* meeting of the SMC). If the SMC review cannot be secured within 24 hours, the study will be halted until SMC review. A decision to proceed or to terminate enrollment, vaccinations, or the trial will be made in consultation with the SMC (including the independent safety monitor), the NIH/NIAID/DMID, and the clinical investigators.

During the study, if the criteria to halt are met, but it is determined that the study may proceed, it is possible that additional events satisfying the same criteria will occur. In this situation, the review process described in the preceding paragraph will be initiated. For example, if two subjects experience events satisfying criterion #3 (below), but upon review the study is

permitted to proceed, only one subject need experience an event satisfying this criterion subsequently to initiate a review described in the preceding paragraph.

If the SMC determines that it is safe to resume the study, but the subject who has already experienced events contributing to another halting rule experiences a SAE, severe AE or laboratory value that is considered by the investigators, ISM, and DMID Medical Monitor to be part of the original reported event, the study will not be halted for this event. If, however, such an event occurs and is considered by the investigators/ISM/DMID Medical Monitor/SMC to be unrelated to the prior event, the study will halt and formal SMC review will occur.

1. If 2 subjects in any single vaccine group experience severe (Grade 3) vaccination site pain/tenderness within 1 week following vaccination;
2. If 2 subjects in any single vaccine group experience moderate (Grade 2 or 3) fever that is associated with the vaccination within 1 week following vaccination;
3. If 2 subjects experience a spontaneously reported adverse event that is graded to be severe (Grade 3) or life-threatening and is associated with vaccination at any time during the follow-up period;
4. If 2 subjects experience a systemic solicited reactogenicity that is graded severe (Grade 3) (other than vaccine-related fever as defined above) within 14 days of vaccination.
5. If 1 subject experiences an SAE judged by an investigator to be associated with vaccination at any point during follow-up;
6. If 2 subjects develop a laboratory value that is considered severe (Grade 3) and that is judged to be associated with vaccination at any point during follow-up;
7. If 1 subject in any vaccine group tests positive for HIV;
8. Any report of new onset of neurologic symptoms. Any other observation occurs that, in the opinion of the PI or NIH/NIAID/DMID, results in a recommendation to halt enrollment and/or further vaccinations.

During clinic visit, the study personnel will ascertain that the reported severity of an AE by the subject meets the predefined criteria of the study of grading the severity of AEs.

If halting rules are triggered and the Internet is not available, then an immediate report will be made by phone and FAX.

9.4 Safety Oversight (Independent Safety Monitor plus SMC)

A qualified and experienced physician not otherwise associated with this protocol will serve as the Independent Safety Monitor (ISM) at the site for this study as per the specifications set forth in the DMID standard operating procedures. The ISM's *curriculum vitae* will be maintained on record. If safety concerns are identified, the ISM may request a meeting of the SMC to review safety data. The ISM will also review all serious adverse events and provide an unbiased written report of the event within 10 calendar days of the initial report. At a minimum, the ISM will comment on the outcomes of the SAE and relationship of the SAE to the study product. The

ISM will also indicate whether he/she concurs with the details of the report provided by the study investigator.

Furthermore, the ISM, a licensed physician, with relevant expertise will have the primary responsibility to provide independent safety monitoring in a timely fashion. The ISM will review SAEs and other adverse events as needed and provide an independent assessment to DMID. The ISM may be a member of the SMC or may not be a member of the SMC but attends the open session of the meetings.

An SMC will be assembled to review the safety data as they are collected. The SMC meets the specifications set forth in the DMID standard operating procedures. The SMC will convene and make recommendations on dosage escalation, based on the safety data collected 14 days after the initial dose of the prior dosage level. The safety data will be compiled by The EMMES Corporation. Based on the recommendations of the SMC, the decision will be made regarding dosage escalation.

***Ad Hoc* Meetings of SMC:** The SMC may convene an *ad hoc* meeting to discuss any issue of safety raised by an investigator, the sponsor, or a member of the SMC. At the discretion of the investigators, the sponsor and SMC members, a non-serious AE that is:

1) Associated with the product
2) Does not meet the stopping rules criteria

may be considered as a trigger for an *ad hoc* SMC meeting to assess the safety of the product, without resulting in halting the enrollment of the trial or further vaccinations.

Furthermore, meetings and roles and responsibilities of SMC are described in study Charter. Please note that an OM will precede the study.

10 CLINICAL MONITORING

10.1 Site Monitoring Plan

Site monitoring will be conducted to ensure that human subject protection, study procedures, laboratory procedures, study intervention administration, and data collection processes are of high quality and meet sponsor, GCP/ICH, and regulatory guidelines, and that the study is conducted in accordance with the protocol and sponsor standard operating procedures. DMID, the sponsoring agency, or its designee will conduct site-monitoring visits as detailed in the monitoring plan or in the Manual of Procedures.

Site visits will be made at standard intervals as defined by DMID and may be made more frequently as directed by DMID. Monitoring visits will include, but are not limited to, review of regulatory files, accountability records, CRFs, informed consent forms, medical and laboratory reports, and protocol compliance. Study monitors will meet with investigators to discuss any problems and actions to be taken and document visit findings and discussions.

11 STATISTICAL CONSIDERATIONS

11.1 Study Overview

The primary objective of the study is to assess the safety and reactogenicity of ascending dosages of Adenovirus Type 35 Based circumsporozoite malaria vaccine among healthy subjects given in 3 intramuscular doses at 0, 1 and 3 months. The secondary objective of the study is to evaluate the immunogenicity of the Adenovirus Type 35 based circumsporozoite malaria vaccine through performance of humoral immune assays (ELISA for Ab vs. CS and Adenovirus Neutralization Assay for neutralizing Ab against Ad35) and cellular immune assays.

The primary outcome measure is the frequency and severity of injection site and systemic AEs. These variables will be analyzed descriptively by:

1. The number of subjects experiencing severe (Grade 3) injection site reactions within 14 days following vaccination.

2. The number of subjects experiencing severe (Grade 3) systemic reactions within 14 days following vaccination.

3. The number of subjects with severe of life-threatening advers reactions considered associated with the vaccination at any point during the study period.

The secondary outcome measures are:

1. Antibody titers against the malaria circumsporozoite antigen at days 0, 28, 56, 84, 112 and 140 (Geometric Mean Titer and individual log ELISA units).
2. Neutralizing antibody titers against Adenovirus type 35 by Adenovirus Neutralization Assay at days 0, 28, 56, 84, 112 and 140.

The exploratory outcome measures are:

1. T cell responses against the malaria circumsporozoite antigen by Elispot.
2. T cell responses against the malaria circumsporozoite antigen by Flow Cytometry
3. Cytokine responses against the malaria circumsporozoite antigen by ELISA after whole blood stimulation.

We propose to confirm the safety of the new vaccine by assessing safety and reactogenicity of the vaccine at 14 days after the initial dose in the lower dosages before escalating to the next

dosage level. Dosage level groups will include 10 subjects given vaccine and 2 subjects given normal saline placebo control intramuscularly.

Sample Size Considerations

The sample size of 10 per group is selected to obtain preliminary safety information on a small cohort of subjects before proceeding to larger trials. If there are adverse events associated with vaccination in this population, this study will have approximately 80% probability to observe at least one such event in a study size of 10 if the true rate is 10%. If there are no adverse events associated with the vaccine, the upper bound for the one-sided 95% confidence interval will be 18% for a study size of 10. The proposed sample size will provide pilot data on secondary outcomes and will not assure adequate power to reject secondary hypothesis of dose-related immunogenic response unless that response is very large.

11.2 Final Analysis Plan

The primary analysis will be conducted on data and samples collected through study day 140 of the last dosage cohort. Once the data are frozen for the primary analysis, subjects will continue to be followed in an unblinded manner for additional SAE surveillance in the Ad35.CS.01 recipients as indicated in the protocol. This additional information will be appended to the study report. This study, like other Phase I studies, is exploratory rather than confirmatory; its purpose is to estimate event rates and patterns of immune responses rather than to test formal statistical hypotheses. Estimates will be presented with their 95% confidence intervals. Descriptive approaches will be used to meet the protocol objectives as stated in this protocol. Results will be presented in tabular format, as well as graphically when appropriate. Formal comparisons between vaccine and placebo groups will not be made. For the purposes of collecting pilot data and planning of potential future trials, some comparisons of secondary outcomes may be made with the control groups, although the study is not powered to detect small to moderate differences.

12 SOURCE DOCUMENTS AND ACCESS TO SOURCE DATA/DOCUMENTS

The site will maintain appropriate medical and research records for this trial, in compliance with ICH E6, Section 4.9 and regulatory and institutional requirements for the protection of confidentiality of subjects. The will permit authorized representatives of the DMID, its designees, and appropriate regulatory agencies to examine (and when required by applicable law, to copy) clinical records for the purposes of quality assurance reviews, audits, and evaluation of the study safety and progress. These representatives will be permitted access to all source data, which include, but are not limited to, hospital records, clinical and office charts, laboratory notes, memoranda, evaluation checklists, pharmacy dispensing records, recorded data from automated instruments, copies or transcriptions certified after verification as being accurate and complete, microfiches, photographic negatives, microfilm or magnetic media, x-rays, and subject files and records kept at the pharmacy, at the laboratories, and medico-technical departments involved in the clinical trial. Forms for use as source documents will be derived from the eCRFs will be provided by the Statistical and Data Coordinating Center (SDCC).

13 QUALITY CONTROL AND QUALITY ASSURANCE

Following written standard operating procedures, the monitors will verify that the clinical trial is conducted and data are generated, documented (recorded), and reported in compliance with the protocol, GCP, and the applicable regulatory requirements. Reports will be submitted to DMID on monitoring activities.

The investigational sites will provide direct access to all trial-related sites, source data/documents, and reports for the purpose of monitoring and auditing by the sponsor, and inspection by local and regulatory authorities.

The Statistical and Data Coordinating Center (SDCC) at The EMMES Corporation will implement quality control procedures beginning with the data entry system and generate data quality control checks that will be run on the database. Any missing data or data anomalies will be communicated to the site(s) for prompt clarification and resolution.

The Quality Management plan will comply with DMID CQMP policy; and the implementation of that plan benefits the internal site audits by

- Supporting substantive performance measurements/findings/corrective actions described in the contract (Reporting Requirements and Other Deliverables),
- provides data to support contract deliverables (1) monthly progress and (2) quarterly technical progress reports.

The plan will be made available at DMID and on site, upon request. Per the contract/statement of work, a clinical research management plan, including quality management, is due within 6 months of the award. Primary site to submit QMP to DMID QMP Reviewers by email. As per the guidance outlined at NIAID/DMID,
http://www3.niaid.nih.gov/research/resources/DMIDClinRsrch/quality.htm

14 ETHICS/PROTECTION OF HUMAN SUBJECTS

14.1 Declaration of Helsinki

The investigator will ensure that this study is conducted in full conformity with the Declaration of Helsinki, or with the ICH GCP regulations and guidelines, whichever affords the greater protection to the subject.

14.2 Institutional Review Board (IRB)

Prior to enrollment of subjects into this trial, the protocol and the informed consent form will be reviewed and approved by the appropriate IRB.

The responsible official for the IRB will sign the IRB letter of approval of the protocol prior to the start of this trial and a copy will be provided to DMID. Notification of the IRB's composition and the institutions Federal Wide Assurance number will be provided to DMID.

Should amendments to the protocol be required, the amendments will be written by the sponsor and provided to the investigator for submission to the IRB.

Subjects will be compensated for their participation in this study. Compensation will be in accordance with the local IRB's policies and procedures, and requires IRB approval.

14.3 Informed Consent Process

The investigator will choose subjects in accordance with the eligibility criteria detailed previously. The investigator will not exercise selectivity so that bias is prevented. All subjects must sign an informed consent form that complies with the requirements of both 21 CFR Part 50 and Health Insurance Portability and Accountability Act (HIPAA) before entering the trial.
A consent form that complies with the requirements of 21 CFR Part 50 will be used.

Prior to the trial, subjects will receive a comprehensive explanation of the proposed vaccine, including the nature and risks of the trial, any known Aes, the investigational status of the components, and the other elements that are part of obtaining proper informed consent. Subjects will also receive a detailed explanation of the proposed use and disclosure of their protected health information, including specifically their biological specimens. Subjects will be allowed sufficient time to consider participation in the trial, after having the nature and risks of the trial explained to them. The consent form must not include any exculpatory statements.

DMID will provide the investigator, in writing, any new information that bears significantly on the subjects' risk to receive the investigational product. This new information will be communicated by the investigator to subjects who consent to participate in the trial in accordance with IRB requirements. The informed consent document will be updated and subjects will be reconsented, if necessary.

Site staff may employ recruitment efforts prior to the subject consenting; however, before any protocol-specific procedures are performed to determine protocol eligibility, an informed consent form must be signed. Subjects will be given a copy of all consent forms that they sign.

By signing the informed consent form, the subject agrees to complete all evaluations required by the trial, unless the subject withdraws voluntarily or is terminated from the trial for any reason.

14.4 Exclusion of Women, Minorities, and Children (Special Populations)

This study will be inclusive of all semi-immune healthy adults who meet the inclusion/exclusion criteria, regardless of religion, gender, or ethnic background. Only individuals who are 18 to 45 years old, inclusive, will be included at this time. Should the outcome of this study be deemed acceptable, additional trials of the vaccine in both younger (infants and children) and older (> 45 years) populations will be initiated.

14.5 Subject Confidentiality

Subjects will have code numbers and will not be identified by name. Subject confidentiality is held strictly in trust by the participating investigators, their staff, the sponsor(s), and their agents. This confidentiality extends to genetic and biological sample tests, in addition to the clinical information relating to participating subjects.

The study protocol, documentation, data, and all other information generated will be held in strict confidence. No information concerning the study or the data will be released to any unauthorized third party without prior written approval of the sponsor.

The study monitor or other authorized representatives of the sponsor may inspect all documents and records required to be maintained by the investigator. This documentation includes, but is not limited to, medical records (office, clinic, or hospital) and pharmacy records for the subjects in this study. Clinical study sites will permit access to such records.

14.6 Study Discontinuation

If the study is discontinued, enrolled subjects will continue to be followed for safety assessments. No further doses of vaccine will be administered.

15 DATA HANDLING AND RECORD KEEPING

The investigator is responsible to ensure the accuracy, completeness, legibility, and timeliness of the data reported.

Forms for use as source documents will be derived from the eCRFs and provided by the DCC to record and maintain data for each subject enrolled in the study. All source documents should be completed in a neat, legible manner to ensure accurate interpretation of data. Black or blue ink is required to ensure clarity of reproduced copies. When making a change or correction, cross out the original entry with a single line and initial and date the change. Do not erase, overwrite, or use correction fluid or tape on the original.

Data reported in the eCRF derived from source documents should be consistent with the source documents or the discrepancies should be explained.

The sponsor will provide guidance to investigators on making corrections to the source documents and eCRFs.

15.1 Data Management Responsibilities

All source documents and laboratory reports must be reviewed by the clinical team and data entry staff, who will ensure that they are accurate and complete. Adverse events must be graded, assessed for seriousness, severity and causal relationship, and reviewed by the site principal investigator or designee. Data collection is the responsibility of the clinical trial staff at the site under the supervision of the site principal investigator. During the study, the investigator must maintain complete and accurate documentation for the study.

The EMMES Corporation will serve as the Statistical and Data Coordinating Center for this study, and will be responsible for data management, quality review, analysis, and reporting of the study data.

15.2 Data Capture Methods

Clinical data (including Aes, concomitant medications, and reactogenicity data) will be entered into The EMMES Corporation's AdvantageEDC^{SM} web-based 21 CFR Part 11-compliant data entry system. The data system includes password protection and internal quality checks, such as automatic range checks to identify data that appear inconsistent, incomplete, or inaccurate. Clinical data will be entered directly from the source documents.

15.3 Types of Data

Data for this study will include safety, laboratory, and outcome measures (*e.g.*, reactogenicity and immunology).

15.4 Timing/Reports

The SMC will convene and make recommendations on dosage escalation, based on the safety data collected 14 days after all subjects at each dosage level cohort have received their first dose. The safety data will be compiled by The EMMES Corporation and the report provided within the agreed upon time frame to complete data entry for distribution to the SMC members. Based on the recommendations of the SMC, the decision will be made regarding dosage escalation.

15.5 Study Records Retention

Records and documents pertaining to the conduct of this study, including CRFs, source documents, consent forms, laboratory test results, and medication inventory records, must be retained by the investigator for at least 2 years following submission of a Biologics License Application or until DMID authorizes transfer or destruction of study records. No study records will be destroyed without prior authorization from NIAID. FDA regulations related to study records retention will also, be followed since this trial is performed under an U.S. IND.

15.6 Protocol Deviations

A protocol deviation is any noncompliance with the clinical trial protocol or Good Clinical Practice (GCP). The noncompliance may be either on the part of the subject, the investigator, or the study site staff. As a result of deviations, corrective actions are to be developed by the site and implemented promptly.

These practices are consistent with Good Clinical Practice:

4.5 Compliance with Protocol, sections 4.5.1, 4.5.2, and 4.5.3
5.1 Quality Assurance and Quality Control, section 5.1.1
5.20 Noncompliance, sections 5.20.1, and 5.20.2.

It is the responsibility of the site to use continuous vigilance to identify and report deviations within 5 working days of identification of the protocol deviation, or within 5 working days of the scheduled Protocol-required activity. All deviations must be promptly reported to DMID, via the EMMES Corporation's AdvantageEDC^{SM}.

All deviations from the Protocol must be addressed in study subject source documents. A completed copy of the DMID Protocol Deviation (PD) Form must be maintained in the Regulatory File, as well as in the subject's source document. Protocol deviations must be sent to the local IRB/IEC per their guidelines. The site PI/study staff is responsible for knowing and adhering to their IRB requirements.

16 PUBLICATION POLICY

Following completion of the study, the investigator may publish the results of this research in a scientific journal. The International Committee of Medical Journal Editors (ICMJE) member journals have adopted a trials-registration policy as a condition for publication. This policy requires that all clinical trials be registered in a public trials registry such as ClinicalTrials.gov, which is sponsored by the National Library of Medicine (NLM). Other biomedical journals are considering adopting similar policies. It is the responsibility of DMID to register this trial in an acceptable registry. Any clinical trial starting enrollment after 01 July 2005 must be registered either on or before the onset of subject enrollment.

The ICMJE defines a clinical trial as any research project that prospectively assigns human subjects to intervention or comparison groups to study the cause-and-effect relationship between a medical intervention and a health outcome. Studies designed for other purposes, such as to study pharmacokinetics or major toxicity (*e.g.*, Phase 1 trials), would be exempt from this policy. This study is of great public health interest. As a result, this study will registered in the NLM registry, ClinicalTrials.gov.

All investigators funded by the NIH must submit or have submitted for them to the National Library of Medicine's PubMed Central an electronic version of their final, peer-reviewed manuscripts upon acceptance for publication, to be made publicly available no later than 12 months after the official date of publication. The NIH Public Access Policy ensures the public has access to the published results of NIH funded research. It requires investigators to submit final peer-reviewed journal manuscripts that arise from NIH funds to the digital archive PubMed Central upon acceptance for publication. Further, the policy stipulates that these papers must be accessible to the public on PubMed Central no later than 12 months after publication.

17 LITERATURE REFERENCES

1. Romero P, Maryanski JL, Corradin G, et al. Cloned cytotoxic T cells recognize an epitope in the circumsporozoite protein and protect against malaria. Nature 1989; 341:323-326.
2. Nussenzweig RS, Vanderberg J, Most H, et al. Protective immunity produced by the injection of x-irradiated sporozoites of plasmodium berghei. Nature 1967;216: 160-162.
3. Clyde DF. Immunization of man against falciparum and vivax malaria by use of attenuated sporozoites. Am J Trop Med Hyg 1975;24:397-401.
4. Rodrigues M, Nussenzweig RS, Zavala F. The relative contribution of antibodies, CD4+ and CD8+ T cells to sporozoite-induced protection against malaria. Immunology 1993;80:1-5.
5. Schofield L, Villaquiran J, Ferreira A, et al. Gamma interferon, CD8+ T cells and antibodies required for immunity to malaria sporozoites. Nature 1987;330:664-666.
6. Weiss WR, Sedegah M, Beaudoin RL, et al. CD8+ T cells (cytotoxic/suppressors) are required for protection in mice immunized with malaria sporozoites. Proc Natl Acad Sci USA 1988;85:573-576.
7. Rodrigues MM, Cordey AS, Arreaza G, et al. CD8+ cytolytic T cell clones derived against the Plasmodium yoelii circumsporozoite protein protect against malaria. Int Immunol 1991;3:579-585.
8. Koram KA, Addae MM, Ocran JC, Adu-Amankwa S, Rogers WO, Nkrumah FK Population based reference intervals for common blood haematological and biochemical paramemeters in the Akuapem North district. *Ghana Med J* 2007 Dec; 41(4):160-166

.

SUPPLEMENTS/APPENDICES

APPENDIX A: SCHEDULE OF EVENTS

**Study Visit**	**1**	**2**	**2A**	**3**	**4**	**5**	**6**	**6A**	**7**	**8**	**9**	**10**	**11**	**11A**	**12**	**13**	**14**	**15**	**16**	**17**	**18**	**19**	**20**
**Study Day**	**Screen[1]**	**0**	**1**	**2**	**7**	**14**	**28**	**29**	**30**	**35**	**42**	**56**	**84**	**85**	**86**	**91**	**98**	**112**	**140**	**196**	**252**	**308**	**365**
Review Inclusion/Exclusion Criteria	X	X					X						X										
Obtain Informed Consent	X																						
Medical History	X	X		X	X	X	X		X	X	X	X	X		X	X	X	X	X	X	X	X	X
Physical Examination[2]	X	X		X	X	X	X		X	X	X	X	X		X	X	X	X	X	X	X	X	X
Vital Signs	X	X		X	X	X	X		X	X	X	X	X		X	X	X	X	X	X	X	X	X
Clinical Safety Laboratory Testing (10 mL blood, urine)	X				X					X						X							
Urine β-HCG[4]	X	X					X						X										
HIV, HCV & HbsAg, (10mL blood)	X																						
Immunogenicity Assays (50 mL blood), thick smear blood slide (days 0, 28 & 84		X					X					X	X					X	X				
Concomitant Medications	X	X	X	X	X	X	X	X	X	X	X	X	X	X	X	X	X	X	X	X	X	X	X
Vaccination		X					X						X										
Assessment of Reactogenicity		X		X	X	X	X		X	X	X	X	X		X	X	X	X	X				
Adverse Event Assessment		X	X	X	X	X	X	X	X	X	X	X	X	X	X	X	X	X	X	X	X	X	X
Home visit for AE assessment[5]			X					X						X									
HIV serology (5 mL blood) risk behavior and SAE assessment																							X

[1]Screening activities must be completed within 42 days prior to Day 0.

[2]A physical exam that assesses lymph nodes, lungs, heart, liver, and spleen will be performed on the screening visit; two and seven days post each vaccination; and thereafter if indicated by the subject's interim medical history. Height and weight will be measured at the screening visit.
[3]Urine pregnancy tests will be required on all females of childbearing potential.
[4]β-HCG: Urine pregnancy tests will be required for all female subjects of childbearing potential and must be within 24 hours prior to vaccination.
[5]Home visits for Days 1-14 following each vaccination to assess AEs except for the scheduled clinic visit days

APPENDIX B: LABORATORY ADVERSE EVENT GRADING SCALE[8]

Parameter	Grade 0<br>"Normal"<br>Screening<br>Values	Grade 1<br>Mild	Grade 2<br>Moderate	Grade 3<br>Severe
Hemoglobin (g/dL)	>10.5 (F)<br>>12.5 (M)	9.5 - 10.5 (F)<br>11 - 12.5 (M)	8.5 - 9.4 (F)<br>10 - 10.9 (M)	≤8.4 (F)<br>≤9.9(M)
WBC (cells/µL)<br>Decreased<br>Increased	 3401<br>8999	 2500 - 3400<br>9000 - 14000	 2000 - 2499<br>14001 - 19000	 <2000<br>≥19001
Platelet count<br>Decreased (per µL)	 ≥125K	 100K - 124K	 50K - 99K	 <50K
AST (IU/L)	≤1 x ULN	>1 - 2.5 x ULN	>2.5 - 4 x ULN	>4 x ULN
ALT (IU/L)	≤1 x ULN	>1 - 2.5 x ULN	>2.5 - 4 x ULN	>4 x ULN
Creatinine (mg/dL)	≤1 x ULN	>1 -1.5 x ULN	>1.5 - 2 x ULN	>2 x ULN
Urinalysis*<br>Protein<br>Blood<br>WBC	 0-trace<br>0-trace<br>0-5	 1+<br>1+<br>6-10	 2+<br>2+<br>11-50	 3+<br>3+<br>>50
Glucose** mg/dl<br>Increased<br>Decreased	65-109	 110-125<br>50-64	 126-140<br>40-49	 >140<br><40
Sodium mEq/L<br>Increased<br>Decreased	 135-148	 149-153<br>131-134	 154-157<br>127-130	 >157<br><127
Potassium mEq/L<br>Increased<br>Decreased	 3.5-5.5	 5.6-5.8<br>3.1-3.4	 5.9-6.2<br>2.7-3.0	 >6.2<br><2.7

For women, results apply only if not menstruating.

* For males and nonmenstruating females.
F = Female
M = Male

ALT = alanine aminotransferase
AST = aspartate aminotransferase
WBC = white blood cell
